# Promoting Smoke-Free Homes Through Biomarker Feedback Documenting Child Exposure to Tobacco Toxins: Protocol for a Randomized Clinical Trial

**DOI:** 10.2196/12654

**Published:** 2019-10-04

**Authors:** Janet Leigh Thomas, Meredith Schreier, Xianghua Luo, Sue Lowry, Deborah Hennrikus, Lawrence An, David W Wetter, Jasjit S Ahluwalia

**Affiliations:** 1 Program in Health Disparities Research Department of Medicine, Division of General Internal Medicine University of Minnesota Minneapolis, MN United States; 2 School of Public Health, Division of Biostatistics Masonic Cancer Center University of Minnesota Minneapolis, MN United States; 3 Clinical and Translational Sciences Institute University of Minnesota Minneapolis, MN United States; 4 Department of Epidemiology University of Minnesota Minneapolis, MN United States; 5 Center for Health Communications Resarch University of Michigan Ann Arbor, MI United States; 6 Center for Health Outcomes and Population Equity Huntsman Cancer Center University of Utah Salt Lake City, UT United States; 7 Brown University School of Public Health and Alpert School of Medicine Providence, RI United States

**Keywords:** biomarker feedback, second hand smoke, randomized clinical trial, cessation

## Abstract

**Background:**

Exposure to secondhand smoke (SHS) early in life increases the risk of sudden infant death syndrome (SIDS), asthma, and respiratory illnesses. Since children’s primary exposure to SHS occurs in the home, these most vulnerable members of our society are not fully protected by recent increases in the adoption of smoking bans in public spaces. Although exposure to SHS is a quickly reversible cause of excess morbidity, few low-income homes strictly enforce smoking restrictions.

**Objective:**

This study aims to test a novel approach to motivate the adoption of home smoking restrictions and to eliminate child SHS exposure by providing parents with objective data documenting home SHS exposure and “biomarker feedback” of child ingestion of tobacco toxins, that is, objective, laboratory-based results of assays performed on child urine, documenting levels of nicotine; cotinine; and NNAL (4-[methylnitrosamino]-1-[3-pyridyl]-1-butanol), which is a metabolite of the known tobacco carcinogen NNK (4-[methylnitro-samino]-1-[3-pyridyl]-1-butanone).

**Methods:**

From 2011 to 2013, 195 low-income, female smokers with children aged ≤10 years residing in their homes were recruited into a two-arm randomized clinical trial. Participants were assigned to one of two groups: biomarker feedback (n=98) and health education (n=97). In-home assessments were administered at baseline, week 16, and week 26. Children’s home SHS exposure and nicotine, cotinine, and NNAL levels from urine samples, measured through a passive nicotine dosimeter and a surface sample of residual tobacco smoke (ie, thirdhand smoke), were collected at all three time points. Primary outcome was dosimeter-verified, self-reported complete home smoking restrictions at 6 months after randomization. Secondary outcomes included parental self-report of smoking behavior change and child urine tobacco toxin (biomarker) change.

**Results:**

Data collection and analyses are complete, and the results are being interpreted.

**Conclusions:**

The study protocol describes the development of a novel community-based controlled trial designed to examine the efficacy of biomarker feedback documenting home and child exposure to SHS on parental smoking behavior change.

**International Registered Report Identifier (IRRID):**

RR1-10.2196/12654

## Introduction

Secondhand smoke (SHS) is a Class A carcinogen with no safe level of exposure. It is estimated that approximately 66% of children aged 3-11 years are exposed to SHS [1]. The human and economic costs of children’s exposure to SHS are staggering and result in 400,000-1 million additional asthma attacks [2], 22,000 asthma-related hospitalizations, 1-3 million outpatient visits due to middle ear disease [2,3], and 100,000-165,000 ear tube operations each year [3,4]. Children exposed to SHS also have higher rates of behavioral and cognitive effects, including attention deficit and hyperactivity disorder [5]. Tobacco use in the home also contributes to 10,000 burn-related outpatient visits and 600 hospitalizations annually [3]. After including increased sudden infant death, exposure to SHS annually contributes to 5000 child deaths and medical costs in excess of US $10 billion [3].

Nearly 19.1 million US children younger than 18 years live in households that have a smoker, making them the most exposed age group [6]. Children are unable to avoid the main source of exposure—often, their close relatives who smoke at home. Furthermore, children have the strongest evidence of harm attributable to SHS [7]. Lower-income children suffer disproportionately from the consequences of SHS exposure, with well-documented higher rates of sudden infant death and asthma [6]. Although the roots of these disparities are complex (eg, poor housing conditions and environmental allergens), exposure to SHS is a prominent and quickly reversible cause of excess morbidity and mortality.

Two reviews detailing the literature on home SHS reduction [8-10] found mixed results. The majority of the studies combined simple self-help materials (ie, instructional pamphlets) with brief intervention (typically one session and as short as 2 minutes) delivered by a nurse or physician. A series of studies of these limited interventions [11-15] reported similar changes in home smoking bans and child’s exposure in intervention as compared to non-intervention control. Compared to the uniform failure of simple self-help or brief SHS reduction interventions, more comprehensive multicomponent interventions have shown more positive effects [16-19]. Results of these trials indicate that multisession, home-based interventions involving motivational counseling might prove more effective. Although the findings for multisession counseling interventions are more promising than self-help or brief interventions, more powerful interventions are clearly needed. Only a few studies have utilized objective laboratory-based assay findings (ie, biomarkers) of children’s exposure to tobacco toxins to reduce home SHS. Initial trials provided this feedback in the form of mailed brochures [20] or brief physicians phone calls to inform parents [21] about the results of biomarker testing on the child and reported null findings. Biomarker feedback seems to be more promising when combined with a more intensive counseling intervention [22,23].

The potential promise of biomarker feedback aimed at reducing home SHS exposure contrasts with a body of literature indicating its limited efficacy on smoking behavior change when used to provide a smoker with objective assay results documenting his/her own exposure to tobacco toxins [24,25]. One possible explanation for the observed discrepancy is that messages that convey risk to a child exposed to parental smoking may be more motivating than messages that convey direct risk to the individual smoker. In their reviews, McClure and Bize [24-26] speculate the other reasons why biomarker feedback to the smoker has not demonstrated a significant impact on behavior change. The first possibility is that the biomarker feedback may not have been sufficiently motivating. This could occur if the risk is perceived to be immutable (ie, a genetic risk factor) or the individual did not understand the meaning of the biomarker on which the feedback was based (ie, meaning of cotinine). Another possibility is that the individual may discount the message because he/she does not trust the source delivering the feedback. A classic line of research demonstrates that attitude change is greater when the communicator is viewed as credible, trustworthy, similar to the recipient, and not trying to change the recipients’ beliefs [27,28]. Finally, elevating the perceived risk of future health consequences (the target of biomarker feedback) alone may be insufficient to bring about behavior change.

Hecht and colleagues [29] documented high levels of one of the most potent tobacco-specific carcinogens—NNAL (4-[methylnitrosamino]-1-[3-pyridyl]-1-butanol—in the urine of children exposed to SHS. This discovery raised substantial concerns within both the scientific and the public health communities, but this information has yet to be utilized to educate parents or caregivers about children’s exposure to cigarette smoke in their homes.

We attended to each of these potential limitations in the design of project STARS (Start Taking Action to Restrict Smoking). STARS is a community-based, randomized trial designed to assess the efficacy of providing culturally sensitive biomarker feedback and objective data about the level of SHS in the home environment to a mother or female caregiver in order to motivate the implementation of home smoking restrictions. Feedback on their child’s exposure to known carcinogens (“cancer causing chemicals”) in tobacco may be more intrinsically motivating than information on other markers of SHS exposure (ie, cotinine). This information was presented by a trusted source (ie, community health workers and a counselor from the participants’ local community) as part of a comprehensive intervention informed by Motivation and Problem Solving (MAPS) [30] counseling designed to empower participants to make positive changes in their own behavior and home environment. In addition, although not included in feedback to parents, we collected and analyzed dust from participants’ homes to examine whether “thirdhand tobacco smoke” (THS) or the residue remaining on surfaces after a cigarette is extinguished could be detected in measurable quantities. The objective of this paper is to detail our approach to building and executing this complex, community-based intervention designed to speed the translation of science from the “bench” to the “community” in order to reduce tobacco toxin exposure among children.

## Methods

### Study Design

**Figure 1:**
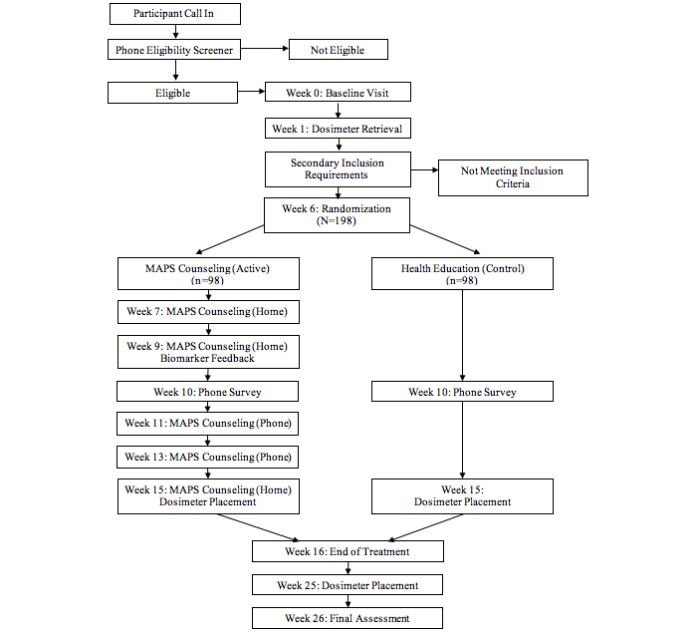
Overview of the study procedures. MAPS: Motivation and Problem Solving.

Figure 1 presents an overview of the study. The primary aim of this two-group, community-based randomized trial (ClinicalTrials.gov NCT01574560) was to assess the efficacy of providing mothers with biomarker feedback documenting their child’s tobacco toxin exposure on reduction of home exposure to tobacco toxins. Once eligibility was determined, participants were randomized to either the intervention arm (biomarker feedback) or the control arm (health education). At baseline, participants in both groups received a series of health education brochures designed to provide information on the dangers of SHS and THS, strategies for managing parenting stress, and tips to assist in a quit attempt. Participants randomized to the active condition were given the results of their child’s urine analyses of nicotine, cotinine, and NNAL as well as the results of the home nicotine dosimeter. They also received five motivational interviewing counseling sessions, approximately 30 minutes in length, spread over 12 weeks. At each counseling session, participants were offered nicotine replacement therapy in the form of gum or lozenge (4 mg). The primary outcome was reduction in home ambient nicotine levels, objectively measured by the passive nicotine dosimeter [31] at 6 months after randomization. Secondary outcomes included child exposure to tobacco toxins measured by urinary levels cotinine, nicotine, and NNAL (a metabolite of NNK, which is a tobacco-specific carcinogen known as 4-[methylnitro-samino]-1-[3-pyridyl]-1-butanone) and a parental report of cigarettes smoked in the home and home smoking policies. Additional aims included identifying potential mediating and moderating effects of demographic variables and psychosocial and tobacco use characteristics.

### Study Setting

The work in this trial was guided by the principles of community-based participatory research (CBPR). CBPR is an approach to research and community engagement that recognizes that the knowledge, expertise, and resources of communities often determine the success of research projects. Thus, we fully engaged our community partner in the research process [32]. Fundamental characteristics of CBPR include collaboration, empowerment, colearning, and community capacity building. An overarching key to the success of CBPR partnerships is the development of authentic partnerships. In project STARS, we partnered with a community health center serving a predominantly lower-income, African American community.

Our selected community health workers were community members chosen by our community partner agency and hired to engage the community in efforts to decrease children’s exposure to tobacco toxins. As liaisons between community members and the research team, they provided cultural mediation and peer social support, advanced culturally appropriate and accessible health education and information, and increased participant access to health services when indicated [33-36]. The approach of using community health workers who are ethnically, socioeconomically, and experientially indigenous to the community has been used in prior cessation trials with success [29,37,38]. Further, evidence of the effectiveness of using community health workers in interventions targeting child health is strong. According to a 2005 Cochrane Review [39-41], community health workers are well positioned to reinforce messages and aid in the protection of children from SHS exposure.

Our community partner provided two full-time community health workers employed by the organization. The community health workers were certified health educators trained to work with individuals who may have difficulty understanding medical providers due to cultural or language barriers. Their role was to recruit, enroll, and conduct all assessment visits. The community health workers were female and indigenous to the target community, adding a source of relatedness and trust for participants. Our community health workers were also trained to interact with participants to enhance autonomy, competence, and relatedness in order to empower self-determined decision making in response to child biomarker feedback. Community health workers are thought to increase the relatedness of the intervention and positively influence message salience and comprehension, clarifying the perceived benefits of adopting home smoking bans.

The research team was located in offices in close proximity to the participant target area. In addition to the two CHWs, the research team was composed of a project coordinator, one full-time counselor from the community, one full-time research assistant, and multiple student workers, many of whom were also from the target community.

### Recruitment

Participants were recruited using widespread efforts and multiple strategies throughout the Twin Cities, Minneapolis and Saint Paul, Minnesota, United States (Table 1). Targeted neighborhoods were identified, and canvassing efforts were carried out in these neighborhoods. Strategies for recruiting participants included attending local health fairs, staff informational sessions, and flyers posted throughout the community. Our primary sources of recruitment were word of mouth (snowball or respondent-driven sampling) and fliers.

The project logo was created by a local community artist and was used on promotional materials. In addition, a wide variety of community services were utilized for recruitment efforts. A partnership was also formed with a local community of churches to assist in church-based recruitment efforts.

**Table 1:** Community-based recruitment source.

Sources of recruitment		Method
Weekly tabloid newspaper, monthly recovery newsletter, papers, African American community		Posted advertisements
Friend, family passed along study information		Provided fliers to participants, encouraged to share
**Health care**		
	Local clinics targeted for uninsured and underserved populations	Posted fliers, closed circuit television advertisements
	Utilization of community partnership with local clinic (medical, mental, and dental services)	Posted fliers, closed circuit television advertisements, service presentation to educate staffed facilitators
Apartment buildings, grocery stores, convenience stores, nail salons, office buildings, bus stations, liquor stores, coffee shops, community hot spots		Posted fliers around the metropolitan area
Community food shelves, Salvation Army		Placed fliers in bags given to consumers
Community centers, women’s resource centers, neighborhood organizations, adoption agency, participation in community events (eg, national night out, health fairs), affiliation with community organizations, Minneapolis Parks		Posted fliers, tabled at hosted events
American Indian Center, Division of Indian Work, American Indian Opportunities Industrialization Center, Minneapolis American Indian Center, Native Community Clinic		Enlisted assistance of member of the community for outreach, posted fliers
Fliers posted in elevators on University of Minnesota campus, campus dental clinic, tobacco research program hotlines		Posted fliers
Employment counselor/agency, workforce centers		Posted fliers
Treatment facilities/centers, detox, transitional housing		Posted fliers
**Education**		
	Child care centers, day care facilities, schools	In service presentations for staff, tabled at parent events
	Head start programs	Tabled at events, placed fliers in child’s bag for taking home
**Media**		
	Facebook, internet, television, Craigslist	Posted advertisements
	Radio	Participated in two radio interviews and ran a public service announcement on a local channel
Church, church group		Partnered with church organization of 12 local churches, provided in-service assistance, paid organization to assist with recruitment
Case worker/manager, child protection worker, public health nurse, nursing agency, Women, Infants and Children clinics, community facilitators		In-service presentations to educate about project to share with clients, posted fliers at agencies

### Participants

Prior to participant enrollment, all study procedures were approved by the Institutional Review Board. Participants called a designated study phone line to be screened for eligibility. All eligibility assessments were conducted over the phone, and initial eligibility status was determined immediately upon completion of the assessment.

**Figure 2:**
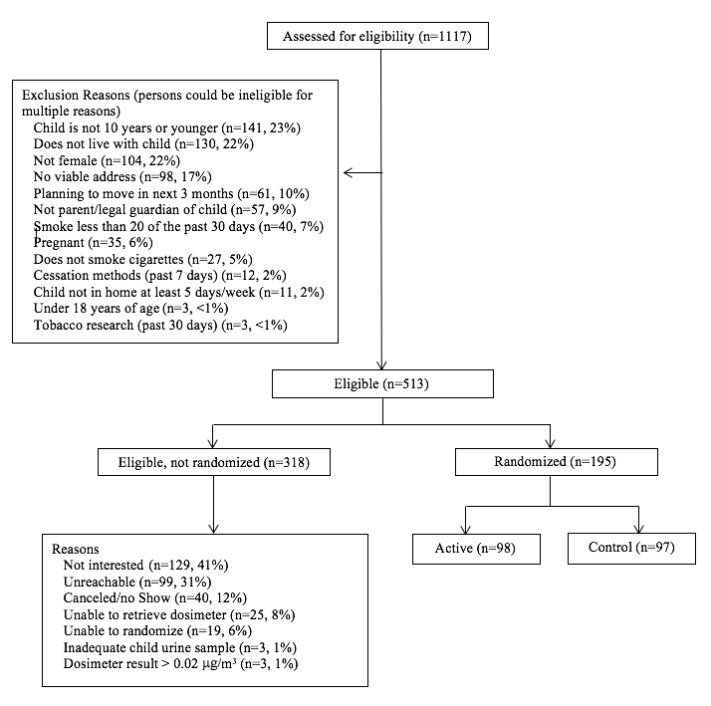
Screening and enrolment of study participants.

A total of 195 participants were enrolled in this study (Figure 2). Recruitment occurred over 21 months, from June 2011 to March 2013. Final follow-up assessments were completed in August 2013. Participants were randomized to either biomarker feedback (active, n=98) or health education (control, n=97) conditions. Eligible participants were female. Although we initially allowed for enrollment of male parents/guardians, we discovered that our counselor did not feel comfortable conducting home visits with a male participant. Participants were at least 18 years of age, not planning to move out of the state over the next 3 months, willing to complete three assessments in their home over a 26-week study period, and the legal guardian of a child aged ≤10 years who lives in the home on at least 5 days of the week. In order to increase the likelihood that the home environment and the child’s urine assay would contain detectible tobacco toxins, participants were eligible if they endorsed smoking cigarettes on ≥20 days in the past 30 days, although they did not have to endorse smoking in the home. Participants were ineligible if they were pregnant, currently using any tobacco cessation aid (eg, counseling or NRT), homeless, living in a shelter or detox facility, living outside of a 30-mile radius of the university, or enrolled in a tobacco research study in the preceding 30-day period.

To determine positive smoking status, participants were asked to provide a urine sample at the baseline visit, which was tested using a NicAlert (Nymox Pharmaceutical Corporation, Quebec, Canada) test strip (a semiquantitative determination of cotinine levels in urine). Individuals with cotinine levels of >10 ng/mL on the NicAlert were verified as active smokers and eligible for study participation. In addition, the child enrolled needed to provide a minimum of 17 mL of urine for sample analysis of nicotine, cotinine, and NNAL. These laboratory assay results were required to provide biomarker feedback to participants in the active condition. If a sufficient amount of urine was not collected at the baseline visit, multiple attempts were made for additional collection visits. The participant was marked ineligible for participation if a sample was not collected from the child within a 2-week window of the baseline visit.

Further, home smoking was verified using a nicotine dosimeter placed in the participant’s home at the baseline visit for a total of 7 days. Upon successful retrieval of the dosimeter, a participant was considered eligible for study participation if their dosimeter value was greater than the limit of detection. The limit of detection for this study was 0.02 μg/m^3^ [42].

### Randomization Process

After final eligibility was determined, participants were randomized into one of two conditions: biomarker feedback or health education. A block randomization with blocks of size 4 was used to improve balance. The statistician generated the randomization numbers for the study using R [computer program] (version R 2.13.0. Austria, Vienna: R Core Development Team), which were imported into and allocated through the Research Electronic Data Capture (REDCap) database. After final eligibility was determined, the assessment staff contacted the project administrator from the participant’s home to ask for the randomization assignment.

### Outcome Assessment Visits

Assessment visits at baseline, week 16, and week 26 were scheduled at the convenience of the participant and were conducted by community health workers at the participant’s home. Visits lasted between 60 and 90 minutes. Staff administered all surveys aloud in an interview format.

Study data were collected via an iPad using a secure, Web-based data collection instrument (REDCap) [43]. At the baseline visit, staff reviewed health education brochures with each participant in a one-time education session. The brochures provided information to participants about the risks of exposure to both SHS and THS, strategies for managing parenting stress, and tips to assist in quitting smoking. A number of additional activities took place at each assessment visit and are detailed below.

#### Urine Collection

Staff collected a urine sample from the youngest potty-trained child in the home. If no children in the home were potty-trained, a urine collection kit was provided to the parent for sample collection. The kit included two all-natural cotton pads, gloves, specimen bags, and an instruction sheet. Participants were instructed to wear gloves to prevent contaminating the sample with nicotine on their hands and to place a pad in the child’s diaper until it was urine soaked. Participants were encouraged to collect a sample overnight, the night before the scheduled visit for heavy soaking, and collect an additional sample the day of the visit. The pad was collected during the visit and transported to our offices where the urine-soaked pad was cut into 0.5-inch wide strips using scissors and placed in a syringe. The syringe was used to extract the urine from the soaked pad. The urine was expelled into a bio specimen cup and stored in a –80°C freezer until it was sent to the laboratory for assaying.

If the identified child was potty-trained, parent assistance was requested to place a specimen commode (aka “hat”) in the toilet for collection and to assist the child in sample collection. Each urine sample was analyzed for the presence of nicotine, a nicotine metabolite (cotinine), and NNAL.

#### Nicotine Dosimeter

At each assessment visit, staff hung a passive air nicotine dosimeter [31], which is an objective, validated method to quantify the level of ambient nicotine present in the air. The dosimeters consist of a filter treated with sodium bisulfate and contained in a 4-cm polystyrene cassette. Nicotine passively diffuses to the dosimeter and binds to the filter. The nicotine dosimeter was hung in the main activity room of each home, as well as the family car, if available. The dosimeter remained in the home and car for a minimum of 7 days for sample collection. After 7 days, staff collected and shipped the dosimeter to the University of California, Berkeley, for analysis using gas chromatography to quantify the presence of nicotine in the air.

#### Residual Tobacco Toxin

Surface dust sampling was collected from two locations—the home and the dashboard of the car—to assess for the presence of THS or residual nicotine and NNK remaining on surfaces after the cigarette was extinguished. A presoaked all-natural cotton wipe was used for surface dust collection. The cotton wipe was soaked with a solution consisting of 1.5 mL of 1% ascorbic acid mixed in reagent-grade water for sample preservation [44]. A 10 × 10 cm wire frame was created for precise marking of the sampling area. The moistened cotton wipe was swabbed over the 100-cm^2^ area on a porous location with visible dust. This sample was taken from the same room that the nicotine dosimeter was placed in as well as another central location in the home.

### Intervention Components

Participants assigned to both the active and control conditions were offered four health education brochures at the baseline assessment. Participants were provided brochures detailing information about SHS, THS (residual smoke exposure), strategies for managing parenting stress, and tips to assist in a quitting smoking attempt.

#### Biomarker Feedback Condition

In addition to the brochures detailed above, participants randomized to the biomarker feedback (active) condition were provided with baseline results from the child’s nicotine, cotinine, and NNAL urine analysis and nicotine levels found in the home and car from the nicotine dosimeter. The information was detailed in a Participant Home Report, and information was verbally reviewed by the counselor. The target behavior in counseling, determined by the participant, was either smoking cessation or the institution of complete home smoking restrictions. Counseling was administered using MAPS counseling techniques [30]. MAPS is a unique approach, derived from a motivational interviewing framework, which utilizes motivationally based techniques to enhance commitment and intrinsic motivation for change in combination with cognitive-behavioral techniques to target self-efficacy, coping, stress, and negative effects. Participants, in collaboration with their counselor, developed a wellness plan that addresses not only smoking cessation or eliminating home smoking, but also other concerns and barriers to changing their smoking behaviors, such as parenting or relationship stress.

#### Counselor Training

All counseling was delivered by a female study counselor who acted as the sole counselor for all participants randomized to the active condition. The study counselor received approximately 40 hours of face-to-face MAPS counseling training, followed by additional training until performance criteria for competence and adherence to the assigned protocol were reached. The counselor participated in monthly, individual supervision to maintain performance standards. In order to monitor protocol fidelity, all counseling sessions were audiotaped, a random sample of two of the counselor’s monthly sessions were coded, and detailed feedback was provided using a modified version of the Motivational Interviewing Treatment Integrity (3.1.1) [45] coding and feedback system to ensure adequate competence and adherence to the protocol.

The counselor followed a strict protocol for completion of the counseling visits and to maximize session adherence/retention. All five sessions were conducted within a 12-week window. The first, second, and fifth sessions were conducted in the participants home, and the third and fourth sessions were conducted over the telephone. The window allowed for sessions to be scheduled every other week until the end of the counseling window. Visits were scheduled at times negotiated between the participant and the counselor. The counselor made daily attempts to reach participants to schedule or reschedule a visit. In addition, contact letters were mailed to the participant’s current address and attempts were made to reach alternate contacts of persons listed by the participant, who would always know how to contact the participant.

#### Health Education Condition

Participants in the comparison condition received health education, which included a review of the aforementioned four health education brochures at the baseline assessment. Participants randomized to the control condition also received the baseline biomarker feedback information but at the completion of their last follow up session, to avoid influencing the study outcomes, but as a gesture of good will and gratitude for participation.

#### Participant Incentives

At each of the three assessment visits, participants were given a choice of receiving a gift card to either a local grocery chain or a discount department store. At baseline, participants received a US $50 gift card and an additional US $5 gift card as compensation for any cell phone usage incurred from study-based calls. Participants were also provided a small gift bag including a manicure kit, a small hair towel, a bath pillow, and a body sponge. Children were given a stuffed bear named Crystal, which was designed and marked by the American Lung Association. The bear included a short poem inscribed on the tag, which read, “Second hand smoke is not a joke! Please keep clear of smoking my dear.” After completing the 16-week home visit, participants received a US $25 gift card, and a US $50 gift card was offered for completion of the 26-week visit.

### Retention

To maximize retention efforts, reminder postcards were mailed to participants a week prior to all visits, and reminder phone calls were made a day before and the day of the scheduled visit. If a visit window was missed throughout study participation, the visit was marked as missed and participant contact was initiated for the next assessment visit. To minimize attrition, staff made periodic reminder calls to participants to reschedule missed visits until the window for completing appointments closed. Participants were asked to provide their home address, home and cell phone numbers, and two alternate numbers to use in the situation that they could not be reached. Participants were also sent a holiday greeting card, child and mother birthday cards, and a thank you card during their six-month participation window.

### Data Management

All data management for this project utilized REDCap [46], a secure Web app for building and managing online surveys and databases. REDCap was used for data entry, data cleaning, identifying any crossovers, and conversion into proper format for data analysis and recoding using SAS [computer program] (Cary, NC: SAS Institute Inc). REDCap was also used to design and administer all assessment surveys. A REDCap tracking feature was also used to follow-up each patient and to prompt staff regarding upcoming data collection points.

### Outcomes

#### Primary and Secondary Outcomes

The primary outcome for this study is change in home SHS exposure measured by the passive air nicotine dosimeter at 26 weeks poststudy enrollment. The specificity of the nicotine dosimeter as a marker of SHS is an appropriate marker to measure a reduction in home air nicotine. Reduction in child’s cotinine and NNAL levels and parental report of smoking behavior change (ie, cessation, quit attempts, reduction, and restrictions) served as secondary outcomes. Specifically, carbon monoxide–verified, 7-day, self-reported point-prevalence abstinence assessed 6 months after study enrollment and defined as no smoking, not even a puff, for 7 consecutive days prior to the final assessment point at month 6 served as the smoking cessation measure [47]. Using a conservative approach, participants who dropped out or were lost to follow-up were considered to be smoking. Adoption of home smoking restrictions was assessed by a question modified from the Current Population Survey Tobacco Use Supplement [48]: “Which statement best describes the rules about smoking in your home?” Participants reported whether there were no bans (“Smoking is permitted anywhere”), some restriction (”Smoking is allowed in some places or at some times”), or complete restriction or ban (“No one is allowed to smoke anywhere”). Smokers were also asked to report the number of cigarettes smoked per day and the number of quit attempts in at least 24 hours since enrollment and their use of programs (eg, Helpline) or pharmacological therapy (eg, nicotine replacement therapy and other cessation medications) at weeks 8 and 26.

#### Treatment Effect Moderators/Mediators

In order to examine psychological mediators and moderators of the proposed intervention (exploratory aims), key factors related to important determinants of smoking behavior change were selected for each set of analyses. These included proposed moderators of effect including sociodemographic variables for parent and child, baseline smoking, and social environmental variables. Participants were asked to detail the number of days they smoked in the last 30 days, the number of cigarettes they smoked on days they smoked, use of smokeless tobacco products and other tobacco used in the last 30 days, whether they used mentholated cigarettes, the number of 24-hour quit attempts they made in the past year, the longest time period they had without smoking a cigarette, and their lifetime nicotine replacement therapy use. Other tobacco-related variables included nicotine dependence, measured using “time to first cigarette” [49] and readiness to quit smoking [50]. Alcohol use [51] (ie, number of days one drank at least one drink, number of days one drank five or more drinks) was also assessed. Social environmental variables included the number of smokers living in the home, number of adult nonsmokers living in the home, outdoor smoking options, location where most smoking occurs, and adoption of home smoking restrictions in the home and car. Child exposure to tobacco smoke was also measured (ie, exposure to tobacco smoke in the home and outside the home in the past week). To assess any potential illnesses or health conditions that might impact the intervention or its effectiveness, parents were asked the following: “Has any health care provider ever told you that your child has any of the following illnesses: (eg, asthma, allergies, cancer, heart problems)?” Psychosocial variables included depressive symptoms (assessed using the 10-item Center for Epidemiological Studies Depression Scale [52]) and stress (measured using the 4-item Perceived Stress Scale [53]). Social influence was measured by asking how many of the participant’s five closest friends smoked cigarettes [54], and social support was measured via the Partner Interaction Questionnaire (Short-Form) [55]. Participants’ view of their social standing in the community and the nation was assessed using the MacArthur Scale of Subjective Social Status [56].

Key mediators are important behaviors, attitudes, and beliefs related to both the proposed intervention and the outcomes. Optimism bias was assessed using a modified version of the Smoking Hazards Scale [57], a 12-item questionnaire with four scales designed to assess perceptions of obvious and subtle health risks due to smoking and other health behaviors. We modified the existing measure to assess perceptions regarding the child’s exposure to SHS. The Passive Smoking Outcomes Expectations Scale [17] was used to assess parents’ expectations of the outcomes that may result from their children’s exposure to SHS. Intrinsic motivation was assessed with the Treatment Self-Regulation Questionnaire [58-60]. We used this tool to measure intrinsic and extrinsic motivation in order to adopt home smoking bans and quit smoking. Smoking abstinence self-efficacy was assessed using the 12-item Smoking Self-Efficacy Questionnaire, and self-efficacy to engage in behaviors to decrease child SHS exposure (eg, adopt home smoking bans) was measured using the Passive Smoking Outcomes Expectations scale [17]. To assess whether receipt of biomarker feedback was associated with changes in motivation and confidence to quit smoking/adopt home smoking bans, participants were asked “How motivated are you to quit smoking/adopt bans?” and “How confident are you in your ability to quit smoking/adopt bans?” In order to assess message salience and comprehension, at the conclusion of each of the three counseling sessions, participants were asked a series of five questions to assess their comprehension and ability to relate to the material presented during that session. Finally, social support for both cessation and adopting home smoking bans was measured using an abbreviated version of the Partner Interaction Questionnaire [61], consisting of six items designed to assess both positive and negative support behaviors [62].

#### Sample Size and Power Considerations

The sample size was chosen to ensure adequate power in order to examine the efficacy of tobacco-specific biomarker feedback in terms of the difference of SHS home exposure (primary outcome) between the two treatment groups at week 26. The power calculation was based on two-sided, two-sample *t* test assuming a type I error of 0.05. A total sample size of 180 (90 per treatment group) was determined to achieve 85% power in order to detect an expected difference (between 1.15 [SD 1.58] μg/m^3^ for the biomarker feedback group and 1.89 [SD 1.68] μg/m^3^ for the health education group) in air nicotine levels measured by dosimeters, which was derived from the assumed adoption rates of home smoking bans of the two treatment groups (55% vs 25%).

### Statistical Analysis

#### Primary Analyses

For the continuous end points such as reduction (ie, change score) in SHS home exposure (primary outcome), child urinary cotinine levels (secondary outcome), and parental self-report amount of smoking, the primary analysis will be the two-sided *t* tests for the measurements at week 26. In case the distribution of nicotine or cotinine level shows nonnormality, the logarithm transformation on the biomarkers or nonparametric Wilcoxon rank sum test will be applied. Categorical variables such as the adoption of home smoking restriction policies and parental quitting behaviors will be analyzed using a Chi-square test (or the Fisher exact test if there are cells with a frequency ≤ 5).

#### Secondary Analyses

Supportive analyses will include multivariable regressions accounting for the effect of relevant baseline sociodemographic, tobacco-related (eg, cigarettes smoked per day), and psychosocial variables (eg, depression), especially if significant effects on the outcomes or imbalance between the study groups are discovered. To utilize the repeated measures data more efficiently, we will also use linear mixed models for the continuous endpoints and the generalized linear mixed models for the binary endpoints. All regression models will include treatment, time, and possibly interactions between treatment and time, after adjusting for other aforementioned covariates. The model-based and empirical estimates of the endpoints for both treatment arms at each of the three time points will be generated and displayed with appropriate tabular and graphical methods.

#### Missing Data Analyses

We will use the intent-to-treat method to include all subjects who were randomized in the final data analysis. For subjects with missing data at week 26, a zero change score will be imputed. As a supplementary analysis, we will also use the completers-only method to analyze variables with missing values.

#### Moderator and Mediator Analyses

Potential treatment effect moderators will be tested by including their main effect and interaction with the treatment group in multivariable regressions. For the analysis of potential mediators, we will employ the four-step logistical process of testing mediational effects developed by Baron and Kenny [63] and others and summarized by Frazier et al [64]. We will also use structural equation modeling techniques to examine the full pattern of predicted mediational pathways. We propose to develop a series of three models to examine how the proposed interventions influence the process of smoking behavior change. The first step in the process is to examine if there is any relationship between the proposed intervention and behavioral determinants as defined by our theoretical model. This step will involve the development of models, with each key behavioral determinant being the dependent variable (Model A). The next model will examine how changes in behavioral determinants are related to interim outcomes, that is, week 16 outcomes (Model B). The final model will examine how changes in behavioral determinants at the interim (16 weeks) evaluation are related to the outcomes at the final evaluation (26 weeks; Model C).

## Results

Ethical approval was received from the University of Minnesota’s Human Research Protection program (Institutional Review Board code number: 0909M72004). Enrollment and analyses for the study are complete, and data interpretation is underway.

## Discussion

Project STARS is one of the first community-based trials to use culturally sensitive biomarker feedback given to the smoking parent or caregiver on their child’s exposure to tobacco toxins. The goal of the study was to reduce SHS exposure in the home, and the intervention was designed to target the smoking mother or female guardian of the child. However, there could be multiple smokers in the home (ie, spouse, partner, or grandparent). Participants were counseled on how to discuss SHS and negotiate home smoking restrictions with others living in the home or visiting the home, but family and social dynamics often complicated the execution of the rules. Further, the majority of our participants lived in areas of high social and economic disadvantage and had competing priorities and additional stressors in their lives that made smoking restrictions difficult. Their desire to create a smoke-free environment for their children was hindered by their physical environment and their responsibilities to care for their children. For example, it was not always plausible to smoke outside of their apartment because the neighborhood was not safe or they could not leave their child alone in the building without supervision. A series of focus groups with smoking mothers in disadvantaged areas found that the need to protect their child’s health was clearly set against competing demands, such as the need to relax and maintain social relationships and the need to be actively present to care for the child and prevent them from harming themselves [65].

Another potential complication was the amount of time the child spent outside of the home. To be considered eligible, the child had to be present in the home 5 days a week, but the number of hours spent in the home was not specified. The child may have spent time in other homes where smoking occurred. Further, children may have ridden in cars, other than their mother’s car, where a driver or passenger was smoking. Although this was assessed in follow-up surveys, it is difficult to obtain an accurate picture of the amount of time the child spent with other smokers. This will be important to keep in mind as we analyze the data from the nicotine badge and the child’s urine assays, as the two sources of measurements may not correlate with each other.

## Appendix

Multimedia Appendix 1 Peer-reviewer report from the National Center on Minority Health and Health Disparities.

## Abbreviations

CBPR: Community-Based Participatory Research
MAPS: Motivation and Problem Solving
REDCap: Research Electronic Data Capture
SHS: secondhand smoke
STARS: Start Taking Action to Restrict Smoking
THS: thirdhand smoke
