# Supplementary material for: Promoting Smoke-Free Homes Through Biomarker Feedback Documenting Child Exposure to Tobacco Toxins: Protocol for a Randomized Clinical Trial
Source: JMIR Res Protoc. 2019 Oct 4;8(10):e12654. doi: 10.2196/12654 (PMC6913685; doi:10.2196/12654)
Supplement: Multimedia Appendix 1 [file resprot_v8i10e12654_app1.pdf]

SUMMARY STATEMENT
(Privileged Communication)

PROGRAM CONTACT:
Nathaniel Stinson
301-594-8704
stinsonn@mail.nih.gov

Release Date: **06/06/2009**

Application Number: **1 P60 MD003422-01**

Principal Investigator

AHLUWALIA, JASJIT S MD

Applicant Organization: UNIVERSITY OF MINNESOTA TWIN CITIES

Review Group: **ZMD1 PA (13)**
National Center on Minority Health and Health Disparities Special Emphasis Panel Centers of Excellence P60

Meeting Date: **03/18/2009** ***RFA/PA:*** **MD08-005**
Council: **MAY 2009** ***PCC:*** **A3**
Requested Start: **07/01/2009**

Project Title: **The Center for Health Disparities Research, Engagement, and Training (CeHDRET)**

SRG Action: **Priority Score: 159**
Human Subjects: 30-Human subjects involved - Certified, no SRG concerns
Animal Subjects: 10-No live vertebrate animals involved for competing appl.
Gender: **1A-Both genders, scientifically acceptable**
Minority: **2A-Only minorities, scientifically acceptable**
Children: **1A-Both Children and Adults, scientifically acceptable**
Clinical Research - not NIH-defined Phase III Trial

Project Year	Direct Costs Requested	Estimated Total Cost
1	949,701	1,408,657
2	927,524	1,389,354
3	949,442	1,461,786
4	949,594	1,464,378
5	944,425	1,455,407
TOTAL	4,720,686	7,179,582

ADMINISTRATIVE BUDGET NOTE: The budget shown is the requested budget and has not been adjusted to reflect any recommendations made by reviewers. If an award is planned, the costs will be calculated by Institute grants management staff based on the recommendations outlined below in the COMMITTEE BUDGET RECOMMENDATIONS section.

1P60MD003422-01 AHLUWALIA, JASJIT

NOTE ABOUT THE SUMMARY STATEMENT: The comments in the CRITIQUE section were prepared before the review meeting by the reviewers assigned to this application and are provided without significant modification or editing by staff. They are included to indicate the range of comments made during the discussion, and may not reflect the final outcome. The RESUME AND SUMMARY OF DISCUSSION section summarizes the final opinion of the committee after the discussion and is the basis for the assigned priority score.

RESUME AND SUMMARY OF DISCUSSION:

Summary of Evaluation Ratings:

Overall Center Score:	159
Research Project 1 Score:	184
Research Project 2 Score:	269

Administrative Core:	Excellent
Community Core:	Outstanding
Res. Training Core:	Excellent
Research Core:	Outstanding/Excellent

This excellent application submitted by Dr. Jasjit Ahluwalia from the University of Minnesota Twin Cities proposes to establish an NCMHD Comprehensive Center of Excellence that focuses on Health Disparities Research, Engagement and Training. The goal of the Center is to create an informed, empowered and active minority community that collaborates with researchers to improve the health of their growing populations. The review panel noted that overall the project is well conceived, goals are significant, the investigator is outstanding, and the assembled team is highly qualified to lead the Center. The environment and the institutional commitment for the proposed Center are considered outstanding. The approach is considered sound, well reasoned, and appropriate to the aims of the project with the support of an adequately developed organizational structure.

The Center will comprise of the four requisite cores, namely, Administrative Core, Community Outreach Core, Research Training Core, and a Research Core with two research projects. The four requisite Cores are rated excellent to outstanding by the review panel.

The Administrative Core is rated excellent for the details it provided, its leadership, organizational structure, support, and the evaluation plans in place to aid success of the proposed Core.

The Community Outreach Core is deemed outstanding for its goals being fostered by detailed, directed activities, and community partnership being fostered by steering committee. In addition, evaluation plans are in place and investigators and consultants will likely contribute to the success of this Core.

The Research Training Core is rated excellent for its great detail about where trainees will come from, ways they will be selected, their mentor selected, and evaluated.

The Research Core is rated outstanding to excellent for being well-organized with several specific initiatives to enhance collaborations on minorities/disparity research in the academic community.

Research Project 1, on minority health (MH), is a randomized intervention trial that will employ Community Health Workers and state-of the-art biomarker feedback to encourage cessation and home smoking restriction among African American families. This project addresses a critical need to eliminate child exposure to environmental tobacco smoke (ETS) in African American homes. Targeting a

concentrated population of refugee immigrants for a randomized behavioral intervention is considered innovative. Although some minor concerns in the study design and approach were noted, this research Project 1 is rated excellent based on the importance of the study problem, potential high significance of the proposed studies, excellent investigator team, and the suitable environment and the research population.

Project 2, on health disparities (HD), is a Medicare claims data analysis regarding disparities in chronic kidney disease in minority populations. The review panel noted that given the disparities in CKD care and progression in African Americans in the United States, the proposed study is very important and unique in its focus. The general approach is sound and appropriate given the study goals and objectives. However, during discussion the reviewers also identified several flaws in the study design which diminished some enthusiasm. Although not uniformly shared, there was also some concern about the adequacy of expertise available on the investigator team for this project 2. Overall level of enthusiasm for Project 2 ranged from good to very good among reviewers.

With regards to the overall center the panel noted that the two projects differ substantially in not only their area of focus (smoking cessation vs. chronic kidney disease) as well as in their approach. In this regard, some reviewers were concerned that without any unifying themes or common shared approaches between the research projects, opportunities for synergy and collaboration will not likely develop to the desired extent. However, not all reviewers shared this concern. After discussion, the panel noted that the proposed Center would highly likely to make a positive contribution to addressing health disparities and that the “whole is greater than the sum of its parts”. Overall, the panel expressed an excellent level of enthusiasm for this application.

DESCRIPRION (Abstract Provided by the Applicant): The long-term goal of the University of Minnesota's (UMN) Center for Health Disparities Research, Engagement and Training (CeHDRET) is to create an informed, empowered and activated community that collaborates with researchers and practitioners to improve the health of their populations. Census data shows the state to be one of the fastest growing states - the total minority population rose from 6.3% in 1990 to 11.8% in 2000. The city of Minneapolis is more than 30% ethnic minorities. The Center will be comprised of four Cores: Administrative, Research, Research Training and Education, and Community Engagement and Outreach. We will support two research projects. Project 1, on minority health (MH), is a randomized intervention trial that will employ Community Health Workers and state-of the- art biomarker feedback to encourage cessation and home smoking restriction among African American families. Project 2, on health disparities (HD), is a Medicare claims data analysis regarding disparities in chronic kidney disease. To maximize the likelihood of the endeavor's success, the Medical School is committing substantial resources to support this initiative: an initial $1 million, annually recurring $300,000, 8,000 sq ft of contiguous UMN office space, and 1,500 sq ft of new space in the North side community for community-based research and programs. The University is committing an additional $100,000 in year 1, increased by the same amount each year, reaching a recurring $400,000 a year, beginning in year 4. The Center will build upon substantial successes we have already accomplished as part of our Program in Health Disparities, including our work in urban health and with African American populations, and will develop community partnerships and, overtime, new research programs with recent African immigrants and refugee populations. The vision is to become a national leader in health disparities and minority health research, serving as a resource for research training, community engagement, and research. Our Center aims are to: 1) Promote HD and MH research at UMN and our partners. 2) Strengthen our community partnerships to be leveraged to improve the health of minorities and new immigrants. 3) Integrate UMN's MH and HD resources into an easy-to-navigate, seamless infrastructure that fosters connections and communication across communities, academic disciplines, and institutional units. 4) Recruit, train and mentor students, fellows, faculty and future research leaders who are from under-represented populations and/or wishing to conduct work in MH and HD.

1A. OVERALL CENTER (PRIORITY SCORE: 159)

CRITIQUE 1 OF THE OVERALL CENTER

Significance: CeHDRET is a timely proposal given the rapidly growing minority populations in Minneapolis metropolitan area and the State of Minnesota. The CeHDRET specifically envision improving the health of new immigrants particularly Somalis through research, research training and community engagement.

Approach: CeHDRET is a well conceived Comprehensive Center of Excellence grant proposal that combines outstanding and experienced investigators with robust institutional commitment to address health disparities through research and research training in the growing minority populations of Minnesota. The center will comprise of the four requisite cores. One of the two special research projects address smoking cessation in African American families with an interventional clinical trial design and the second project is a secondary data analysis focusing on chronic kidney disease in minority populations. CeHDRET will recruit and train (with substantial institutional resources) new fellows and post-graduate trainees of minority background who are interested in pursuing research on minority health and health disparities. Institutional funds will also be utilized to recruit five new faculty members to work on health disparities research. A pilot grant program will be implemented and geared towards trainees and junior investigators. The organizational structure described the proposed CeHDRET as a center that reports directly to the Dean of the Medical School. A comprehensive evaluation plan is proposed with well defined milestones.

Innovation: Targeting a concentrated population of refugee immigrants for a randomized behavioral intervention is innovative. Several operational concepts within the CeHDRET are also new and likely to broaden the scope and skills that can be used to conduct health disparities research and community engagement activities

Investigators: The Principal Investigator – Jasjit Ahluwalia, MD, MPH, MS is a highly qualified and remarkably suited to lead the center. Dr. Ahluwalia is the Associate Dean for Clinical Research in the Medical, and Professor of Medicine and Professor of Epidemiology and Community Health. He was trained in internal medicine, public health and health policy. He has nearly two decades of experience in conducting research in underserved and minority groups and has an impressive record of NIH funding including five large randomized clinical trials. The Center Co-Director, Dr. Kolawole Okuyemi is also well qualified. Dr. Okuyemi is the Director for the Program in Health Disparities Research and Associate Professor of Family and Community Medicine. He has a strong record on NIH funding including an R01 to conduct a clinical trial titled " Improving Nicotine Replacement Therapy Adherence and Outcomes in Homeless Smokers" The core directors are well qualified.

Environment: The environment at the University of Minnesota is superbly suited for the CeHDRET. Ample resources and expertise are available and the institutional commitment is tremendous totaling approximately $3 million in direct cash transfer and 9500 sq ft of new space.

Additional Review Criteria:

Human Subjects: Applicable (G1, M2, C1)

Risks and Protections: Acceptable

Inclusion of Women: Acceptable: G1A

Inclusion of Minorities: Acceptable; M2A

Inclusion of Children: Acceptable: C1A

Targeted Recruitment for Clinical Trials: Acceptable

Vertebrate Animals: Not Applicable

Animal Care and Use: Not Applicable

Budget Recommendations: The requested budget is appropriate.

Administrative Comments: None

CRITIQUE 2 OF THE OVERALL CENTER

Significance: The population of minorities and minority immigrants is growing in the Twin Cities. The Center will foster integration among the University, Medical School, and Community, and will support second and third generation research (i.e., to understand and identify sources for observed disparities, and develop interventions to reduce disparities, respectively). The CeHDRET aims to become a national leader in health disparities and minority research and serve as a resource for research, research training, and community engagement.

Approach: The CeHDRET mission, to improve minority health and reduce health disparities through the integrated cores and 2 research projects, builds upon the many impressive commitments the University and Medical School have made to increasing diversity among students and faculty, as well as fostering community partnerships. The Center has depth in its research and dissemination focus (on African American and African immigrant populations) and breadth in its coalition building activities, speaker events (CE/OC), education and training (RT/EC), support of pilot studies (RC), and Regional Capstone Conference (AC).

Innovation: Housing the Center in the Community it serves should have good impact for partnerships. It is located next to the Office of Clinical Research and the Health Survey Research Center which should also have good impact for research.

Investigators: The PI will be Director of the Administrative Core and Co-Director of the RT/E Core. Until recently he was the Executive Director of the Office of Clinical Research, and is presently Professor of Epidemiology and Public Health, and Associate Dean for Clinical Research in the Medical School. His research experience and funding in the domain of public health, and mentoring of students and faculty in the area of diversity also suggest he will be successful as this COE Director.

Environment: There are ample University resources, both physical and structural (e.g., The Program in Health Disparities Research, The School of Public Health includes a Health Disparities Work Group which developed an approved Health Disparities Interdisciplinary Concentration available to MPH students), and functional (e.g., existing community/scientist partnerships) to focus Center activities. Both the University and the Medical School have committed funds for this effort (1.4 million and 2.5 million, respectively) to improve minority health and reduce health disparities. Their continuing efforts to build diversity complement the focus of this Center, which would serve as a hub for focusing research, training/education, and community outreach activities. Data sharing strategies (within and outside the University) are in place. There is much University, Medical School, and Community partnership support as evidenced in letters of support and in financial support.

Renewal: NA Resubmitted: NA

Human Subjects: Applicable

Risks and Protections: Acceptable

Inclusion of women, minorities, and children: Acceptable

Targeted Recruitment for Clinical Trials: Acceptable

Vertebrate Animals: NA

Animal Care and Use: NA

Budget Recommendations: As requested

CRITIQUE 3 OF THE OVERALL CENTER

Significance: The long-term goal of the University of Minnesota's (UMN) Center for Health Disparities Research, Engagement and Training (CeHDRET) is to create an informed, empowered and activated community that collaborates with researchers and practitioners to improve the health of their populations. The Center will be comprised of four Cores: Administrative, Research, Research Training and Education, and Community Engagement and Outreach. Two research projects are proposed. Project 1, on minority health (MH), is a randomized intervention trial that will employ Community Health Workers and state-of the-art biomarker feedback to encourage cessation and home smoking restriction among African American families. Project 2, on health disparities (HD), is a Medicare claims data analysis regarding disparities in chronic kidney disease.

Approach: The approach is very sound. The arrangements and organizational structure are adequately developed, well reasoned and appropriate to the aims of the project. The PI and Director is Jasjit S. Ahluwalia, M.D., M.P.H.; Deputy Director and Director of the Community Engagement and Outreach Core is Kolawole S. Okuyemi, M.D., M.P.H.; Director of the Research Training and Education Core is Selwyn M. Vickers, M.D.; Director of the Research Core is Susan A. Everson-Rose, Ph.D.; Co-Director of the Research Core is Mary Story, Ph.D.; and, Co-Director of the Community Engagement and Outreach Core is Kathleen T. Call, Ph.D. This center will: 1) Promote HD and MH research at UMN . 2) Strengthen community partnerships to be leveraged to improve the health of minorities and new immigrants. 3) Integrate UMN's MH and HD resources into an easy-to-navigate, seamless infrastructure that fosters connections and communication across communities, academic disciplines, and institutional units. 4) Recruit, train and mentor students, fellows, faculty and future research leaders who are from under represented populations and/or wishing to conduct work in MH and HD.

Investigators: The Center will consists of administrators, researchers, educators and community advocates that spans across six the colleges and multiples departments at the MUSC
Jasjit S. Ahluwalia, M.D., M.P.H., M.S. - Associate Dean for Clinical Research in the Medical School and Professor of Medicine and Professor of Epidemiology and Community Health, will serve as Director of the Administrative Core and the CeHDRET and Principal Investigator of the award. Dr. Ahluwalia will oversee all aspects of the Center, working with University leadership to develop a home for investigators conducting research in minority health and health disparities. By working closely with University leadership, reporting directly to Deborah E. Powell, M.D., the Medical School Dean and Vice President for Clinical Sciences, Dr. Ahluwalia will advocate for high visibility and representation of CeHDRET issues at the highest level within the University. He will work closely with the five Deans of the Academic Health Center colleges and schools, as well as Department and Division heads to recruit and retain clinical and translational investigators from underrepresented backgrounds and/or conducting minority health research. He will actively drive the mission of CeHDRET by ensuring it remains high among the priorities of the Institution. He will serve as a mentor to junior faculty, students, and other trainees. He will oversee prioritization of Center resources; both funds awarded through this application and the considerable institutional funds that have been secured. He will chair the Executive Committee, and the three Core Directors and the Administrative Director will report directly to him.
Kolawole S. Okuyemi, M.D., M.P.H. - Director for the Program in Health Disparities Research and Associate Professor of Family Medicine and Community Health at the University of Minnesota Medical School (0.60 calendar months), will serve as Deputy Director for the Administrative Core and of the CeHDRET. For the past ten years, Dr. Okuyemi has worked with minority and underserved populations to eliminate health disparities and improve minority health. Dr. Okuyemi has extensive experience in

community engagement among minority and underserved populations around initiatives to eliminate health disparities. He will assist the Director in all activities and will Chair the Executive Committee meetings in the Director's absence. His primary role will be as the point person for all non-University activities.

Eileen M. Harwood, Ph.D. - Assistant Professor in the School of Public Health, Division of Epidemiology and Community Health (1.20 calendar months), will serve as Evaluation Director within the Administrative Core and of the CeHDRET.

Environment: The institutional commitment to the pursuit of minority health and health disparities research, including provision of resources, administrative authority and recognition is excellent. To maximize the likelihood of the endeavor's success, the UMN Medical School will commit substantial new resources to support this initiative. This commitment will include: 1) a start-up fund of $1 million; 2) a recurring fund of $300,000 a year; 3) 8,000 sq ft of contiguous University office space, and 1,500 sq ft of new space in the North side community for community-based research and programs and 4) the University is making a commitment of $100,000, increased each year by the same amount, to a total recurring amount of $400,000 a year (beginning in year 4). The scientific environment(s) in which the work will be done will contribute to the probability of success. The proposed studies will benefit from unique features of the scientific environment(s), and easy access to subject populations institutional support is evidence.

Human Subjects:
Risks and Protections: Acceptable

Inclusion of Women: Acceptable/ Gender Code-G

Inclusion of Minorities: Acceptable/ Minority Code-M

Inclusion of Children: Acceptable/ Children Code- C

Targeted Recruitment for Clinical Trials: Acceptable

Budget Recommendations: Appropriate

Administrative Comments: one main concern is the time commitment of the PI

CRITIQUE 4 OF THE OVERALL CENTER

Significance: The research goals of the Center address two important problems which affect African American health: chronic kidney disease and tobacco use. The two projects differ substantially in not only their area of focus (smoking cessation vs. chronic kidney disease) but, perhaps more importantly, in their approach. In fact, it is difficult to discern any unifying theme between the two projects except African American health most generally. Without any unifying or common shared approaches or themes, it is unclear what opportunities for synergy and collaboration will develop.

Approach: Again, because the aims, areas, and approaches in the two projects differ so widely, the potential for synergism between the two main projects is limited. Synergism between the community outreach project on smoking cessation and Project 1, a community-based biofeedback intervention to reduce ETS exposure among children, may be possible. The second project, with its focus on data analysis from a national database of medicare claims, seems isolated from the rest of the Center's activities. There is little to no opportunity, for example, for involvement/contributions from or to the community outreach core or the research and education core and project 2. It is unclear why it is proposed as a project in this application, rather than a separate research project. On the positive side, the Center's commitment to encouraging and fostering new investigators, even in the absence of NIH-

funded pilot grant program, is impressive and shows the extent of institutional commitment to the Center.

Innovation: The specific project ideas are not particularly unique or innovative. The most innovative part of the application may be the community outreach core, whose activities (though not research per se) involve community organizations in a substantial way to address subjects of expressed concern and relevance to community members.

Investigators: The investigators are almost all extremely well-qualified and experienced in minority and disparities research.

Environment: Institutional support is impressive: two pilot grant programs and recruitment of 5 new faculty; total of almost $3 million over 5 years from both Medical school and university is pledged. University of Minnesota has invested substantial resources in the Center for Health Disparities that is already established and productive.

Minnesota has a relatively low percent of minorities (11.8%, as compared to 26% nationally). Although this would argue against the establishment of a NCMHD Center of Excellence in Minneapolis, it seems likely that the coasts are over-represented and having a mid-western presence may be advantageous. Also, the minority population of Minnesota is growing.

Renewal: Comment on Progress Report: Not applicable

Resubmitted: Comment on Improvement: Not applicable

Additional Review Criteria:

Human Subjects: Applicable

Risks and Protections: Acceptable

Inclusion of Women: Acceptable

Inclusion of Minorities: Acceptable

Inclusion of Children: Acceptable

Targeted Recruitment for Clinical Trials: Acceptable

Vertebrate Animals: Not Applicable

Budget Recommendations: As requested

Administrative Comments: None

1B. ADMINISTRATIVE CORE: (SCORE DESCRIPTOR: EXCELLENT)

CORE LEADER (S): AHLUWALIA, JASJIT

DESCRIPTION (PROVIDED BT THE APPLICANT): The Administrative Core will serve as the primary organizing structure for the University of Minnesota Center for Health Disparities Research, Engagement, and Training (CeHDRET). Upon receipt of the Center of Excellence P60 award, the planned activities will be seamlessly integrated with the Medical School's Program in Health Disparities. The Administrative Core will: 1) Coordinate the Research Training, Community Engagement, and Research Cores, and at a high level,

the two Research Projects; 2) Manage operations; 3) Coordinate disparities activities across the University; 4) Promote the Center's Activities; 5) Organize and staff annual strategic retreats of Center leadership and members; 6) Recruit and retain minority faculty and faculty conducting health disparities and minority health research; 7) Facilitate communication; and, 8) Conduct evaluation activities of the Center. The Principal Investigator (and Administrative Core Director) and Executive Director, Dr. Ahluwalia, reports to Deborah Powell, the Medical School Dean. The Director will have substantial authority, responsibility, and resources and will lead the allocation of the University and community space, as well as the NIH P60 and institutional funds. The leadership team will also include Kola Okuyemi, MD (Deputy Director and Director of the Community Engagement Core), Selwyn Vickers, MD (Director of the Research Training Core and past PI of the UAB EXPORT), Susan Everson-Rose, PhD (Director of the Research Core), Mary Story, PhD (Co-Director of the Research Core), and Kathleen Call, PhD (Co-Director of the Community Engagement Core. The Center will have three Boards. An External Advisory Board comprised of seven members of national prominence who have research expertise, gender, and ethnic diversity. The Internal Advisory Board will be comprised of eight institutional leaders; six Deans, the Vice President for Equity and Diversity, and the Director of our Comprehensive Cancer Center. Finally, the Community Collaborative Board will include 10 members from various sectors of the Twin Cities, and one member each from our two lead Community Partners. Consistent with our philosophy of inclusivity, Center membership will be open to all from the University, our partner educational institutions, community and corporate partners, and local practitioners.

CRITIQUE 1 OF THE ADMINISTRATIVE CORE

Approach: This Core serves as the primary organizing structure for the other Cores, manage operations, coordinate disparities activities across the University, promote Center activities, organize and conduct annual strategic retreats of Center leadership and members, recruit and retain diverse faculty focusing on health disparities and minority health research, facilitate communication (including conflict mediation), and conduct activity evaluation. This Core’s activities will be integrated with activities in the Medical School’s Program in Health Disparities. Disparities activities include: developing initiatives (grant-writing, research workshops), annual Accelerate the Future symposium (highlighting work of community partners), Year 5 Midwest Regional Conference on Health Disparities and Minority Health (a 2-day mid-western region conference particularly for other COE P60 grantees, national keynote speaker), disseminate a monthly newsletter, Evaluate (through meeting minutes and meeting of goals from Center concept model) and replace leadership (if necessary), evaluate Center and Cores.

Investigator: The PI and Executive Director is Associate Dean for Clinical Research in the Medical School and Professor of Medicine and of Epidemiology and Community Health. As founding Executive Director of the Office of Clinical Research he led the clinical and translational research program across the University’s six health sciences schools and inter-professional centers and institutions and was liaison for research to clinical affiliates. He will report to Dean Deborah Powell, UMMS. The Deputy Director (and Director of the CE/O Core) is the Director for the Program in Health Disparities Research and Associate Professor of Family Medicine and Community Health at the Medical School. The efforts for this Center should integrate relatively seamlessly with these efforts. Leadership also includes Directors of the other Cores. In addition, the Evaluation Director will evaluate all components of CeHDRET, she is an Assistant Professor in the School of Public Health, Div of Epidemiology and Community Health. In addition to the executive committee, which is comprised of the Core Directors, 3 Boards are proposed: an External Advisory Board, with well qualified minority health scientists and administrators, is in place (5 letters of support included; institutional letter of support from Dean Powell, 6th member) and is partly funded by the medical school (years 3 and 4), an Internal Advisory Board, comprised of 8 institutional leaders with relevant experience in minority health (6 Deans, the VP for Equity and Diversity, and the Director of the Comprehensive Cancer Center; 5 Deans and other letters of support included), and the Community Collaborative Board (10 community supporters, 2 lead community partners; letters of support included). The Executive Committee will meet bi-weekly and the Advisory Boards will meet annually, the Collaborative Board will meet bi-monthly.

Environment: There is excellent institutional and community support for the proposed activities. The detail with which duties are outlined ensures that the Center will have good focus to meet its goals.

Budget recommendations: The budget is commensurate with personnel requirements and proposed activities.

CRITIQUE 2 OF THE ADMINISTRATIVE CORE

Approach: The Administrative Core will serve as the primary organizing structure for the University of Minnesota for Health Disparities Research, Engagement, and Training (CeHDRET). The arrangements and organizational structure are adequately developed, well reasoned and appropriate to the aims of the center. The application, also describes how day-to-day management will be accomplished. The Administrative Core will: 1) Coordinate the Research Training, Community Engagement, and Research Cores, and at a high level, the two Research Projects; 2) Manage operations; 3) Coordinate disparities activities across the University; 4) Promote the Center's Activities; 5) Organize and staff annual treats of Center leadership and members; 6) Recruit and retain minority faculty and faculty conducting health disparities and minority health research; 7) Facilitate communication; and, 8) Conduct evaluation activities of the Center.

Approach: The Principal Investigator (and Administrative Core Director) and Executive Director, Dr. Ahluwalia, reports to Deborah Powell, the Medical School Dean. The Director will have substantial authority, responsibility, and resources and will lead the allocation of the University and community space, as well as the NIH P60 and institutional funds. The leadership team will also include Kola Okuyemi, MD (Deputy Director and Director of the Community Engagement Core), Selwyn Vickers, MD (Director of the Research Training Core and past PI of the UAB EXPORT), Susan Everson-Rose, PhD (Director of the Research Core), Mary Story, PhD (Co-Director of the Research Core), and Kathleen Call, PhD (Co-Director of the Community Engagement Core. The Center will have three Boards. An External Advisory Board comprised of seven members of national prominence who have research expertise, gender, and ethnic diversity. The Internal Advisory Board will be comprised of eight institutional leaders; six Deans, the Vice President for Equity and Diversity, and the Director of our Comprehensive Cancer Center. Finally, the Community Collaborative Board will include 10members from various sectors of the Twin Cities, and one member each from our two lead Community Partners. The Administrative Core builds upon active community/academic partnerships with a demonstrated commitment to provide health outreach to vulnerable populations and research, training and educational opportunities to populations most affected by health disparities. The Administrative Core is very well described and has excellent structure. The long-term Management is not very well articulated.

Investigators: The qualifications, experience, and administrative competence of the Administrative Core Director are appropriate. The individuals providing key management and decision-making comprise a uniquely synergistic blend of critical skills and disciplinary expertise to develop and implement innovative approaches to prevailing problems embedded in health disparities. Jasjit S. Ahluwalia, M.D., M.P.H., M.S. - Associate Dean and Professor of Medicine and Professor of Epidemiology and Community Health, will serve as Director of the Administrative Core and the CeHDRET and Principal Investigator of the award.

Environment: The institutional commitment is evident to support minority health and health disparities research and other proposed activities, including provision of resources, and administrative authority. There is a firm institutional commitment for space to accommodate the dedicated office personnel.

Budget: Appropriate

CRITIQUE 3 OF THE ADMINISTRATIVE CORE

Approach: The Administrative Core is directed by the CeHDRET PI – Dr. Ahluwalia. The Center Co-Director – Dr. Kolawole Okuyemi will serve as Deputy Director and Dr. Eileen Harwood (Assistant Professor of in the School of Public Health) will serve as the Evaluation Director. An Administrative Director at 0.50 FTE is yet to be named. Other key personnel in this core include a Web Master and a Program Manager. Eight specific aims (functions) are defined for the core. The core will be located in the Medical School although note is made that it will be a highly visible institution-wide resource. Governance is well delineated with the Director reporting to the Medical School Dean. An Executive Committee comprising the PI and core directors will responsible for the Center's operation. Provision is made for an External Advisory Committee of seven members from different institutions. Eight institutional leaders including the Deans of the Medical, Nursing, Dentistry, Public Health and Pharmacy will provide advice to the Center on issues of strategic planning. A 10-member Community Collaborative Board is also proposed. The composition of the various committees and their functions are well conceived and adequately defined. An excessive number of meetings is proposed for the Community Collaborative Board (6 times/year). A comprehensive evaluation plan is presented based on the Logic Model.

Investigator: The investigators are well qualified.

Environment: The environment is well resourced and more than adequate.

Budget Recommendations: The budget is appropriate

Administrative Comments: None

CRITIQUE 4 OF THE ADMINISTRATIVE CORE

Approach: Executive committee (PIs and core directors) will meet bi-weekly. External advisory board will meet annually. Internal advisory board will meet annually: primarily deans of the school of public health, pharmacy, nursing, dentistry, and medical schools. Community Collaborative Board will meet every 2 months.

Investigator: Directors Dr. Ahluwalia with Dr Harwood (evaluation director) and Dr. Okuyemi (Deputy director). Dr. Ahluwalia will devote 25% effort to the administrative core, which is appropriate.

Environment: Strong financial commitment from University/Medical School.

Budget Recommendations: Appropriate.

Administrative Comments: None

1C. COMMUNITY ENGAGEMENT/OUTREACH CORE (SCORE DESCRIPTOR: OUTSTANDING)

CORE LEADER (S): OKUYEMI, KOLAWOLE

DESCRIPTION (PROVIDED BY THE APPLICANT): The primary objective of the Community Engagement & Outreach Core of the proposed Center for Health Disparities, Research, Engagement, and Training (CeHDRET) is to engage African American and African immigrant communities in the Twin Cities of Minneapolis and St. Paul, Minnesota, in efforts to improve minority health and reduce health disparities. The Community Engagement & Outreach Core has two specific aims: Aim 1: To establish a community-academic partnership that will provide a unifying and transformative organizational framework for improving minority health in the Twin Cities of Minnesota; Aim 2: To engage community partners from the local African American and African immigrant (Somali) communities in the dissemination of interventions to promote fruit and vegetable consumption and reduce tobacco use. Core activities, guided by the principles of community-based participatory research (CBPR), will be a collaborative effort among the University of Minnesota, the

Stairstep Foundation (which has a coalition of 32 African American churches in the Twin Cities), and the Minnesota International Health Volunteers (MIHV). For Aim 1, we will focus on the five key phases that define CBPR: partnership building, capacity building, needs assessment, community action plan (CAP) development, and CAP implementation and evaluation. For Aim 2, we propose two activities. First, we will partner with the Stairstep Foundation to disseminate Body & Soul, an evidence-based program to promote fruits and vegetables consumption in African Americans. Second, we will partner with MIHV to develop a culturally-targeted community-based intervention to reduce tobacco use in the Somali community. We also will work closely with the CeHDRET Research Training and Education Core to engage trainees in community research and outreach activities. Through partnership and capacity building, we plan to work collaboratively to create mutually beneficial and sustainable programs with the goal of producing knowledge that may be directly applied to improving community health. Along with our community partners, we plan to develop, implement, and evaluate innovative community engagement and outreach programs with the ultimate goal of reducing health disparities.

CRITIQUE 1 OF THE COMMUNITY ENGAGEMENT/OUTREACH CORE

Significance: The objective is to engage minority communities in health promotion programs to reduce their disproportionate cancer and cardiovascular risk and reduce cancer and cardiovascular risk factors. The long-term goal of the Center for Health Disparities Research, Engagement, and Training (CeHDRET) is to create an informed, empowered, and activated community and research/practitioner base to collaborate on improving minority health. The Center aims to bridge University scientists and partner institutions and community members by removing barriers and incentivizing collaboration opportunities. This builds upon other programs in place at the University, which serve as models of success, e.g., the Urban Outreach/Engagement Center, Program in Health Disparities Research Community Collaborative Pilot Grants where university scientists and community partner are Co-Investigators.

Approach: Establish community-academic partnership to provide unifying and transformative organizational framework for improving minority health in the Twin Cities, engage community partners from local African American and African immigrant communities to disseminate interventions to promote fruit and vegetable consumption and reduce tobacco use. Guided by CBPR (community-based participatory research) principles, the collaborative effort among the University of Minnesota, the Stairstep Foundation (coalition of 32 African American churches) and the Minnesota International Health Volunteers is to develop, implement, and evaluative innovative community engagement and outreach with the goal of reducing health disparities. Partnering with SF, this Core will disseminate Body & Soul, an evidence-based program to promote fruit and vegetable consumption, and partner with MIHV to develop a targeted intervention to reduce tobacco use. This Core will work closely with the Research Training and Education Core to engage trainees in community research and outreach.

A 9-member Steering Committee will convene with 3 members from each SF, MIHV, and UM. They will develop a community action plan that drives the focus of research efforts based on community needs. There is a Community Collaborative Board in place (letters of support included), which will provide ongoing guidance to the steering committee, and there will be Community Dialogue Sessions (monthly) and Distinguished Scholar Series meetings (quarterly). A community partner assessment will evaluate the community's experience with the partnership.

Investigator: Core Director is presently Director for the Program in Health Disparities Research and Associate Professor of Family Medicine at UMMS, where he leads an interdisciplinary team of scientists and trainees. He has a decade-long program of research on minority health and interventions targeting health disparities and has mentored minority students and faculty in this domain. The Co-Director, as Associate Professor in the School of Public Health, is Co-Chair of the School's Health Disparities Work Group. The goals of this Group include creating long-lasting partnerships with SPH faculty and the community, strengthen collaborations, and ensure health disparities is integrated into the curriculum. She has conducted a number of CBPR projects over the years and has mentored

trainees (under/graduate/postdoctoral). There is also a Program Manager to help direct University community engagement initiatives, with which he already has a lot of experience. A consultant who is expert in conducting health promotion interventions for special populations will consult to review the Core annual.

The University and Medical School are well-equipped to support this work and have committed monies to support the Center. There are several programs currently in place (UROC, PHDRC – see above) that would benefit from these efforts. It is not clear that Center efforts (as described by the Cores) duplicate other ongoing initiatives. It does appear that the Center efforts would complement and strengthen existing structures that are functioning at the University and in the community.

Budget recommendations: Commensurate with the personnel, resources, and activities proposed. Community Organization Support is sought for the main community partners (mentioned above) to cover expenses related to the partnership. Financial support for the Body and Soul program (a group of African American churches) is included. There are consortium agreements with NCI's CIS and MIHV regarding these efforts (agreements included with materials) and there is a memorandum of understanding in place for the main community partners (SF and MIHV, who each receive $25K per year to implement proposed activities in the manner they see fit. The expectations and deliverables are outlined in the memoranda of understanding (included with the application materials).

Human Subject Code: 30

CRITIQUE 2 OF THE COMMUNITY ENGAGEMENT/OUTREACH CORE

Significance: The proposed goals and activities of the community engagement/outreach core have a clear potential to the translating research findings to the community. The primary objective of the Core is to engage African American and African immigrant communities in the Twin Cities of Minneapolis and St. Paul, Minnesota, in efforts to improve minority health and reduce health disparities. The Core has two specific aims: Aim 1: To establish a community-academic partnership that will provide a unifying and transformative organizational framework for improving minority health in the Twin Cities of Minnesota; Aim 2: To engage community partners from the local African American and African immigrant (Somali) communities in the dissemination of interventions to promote fruit and vegetable consumption and reduce tobacco use. Core activities, guided by the principles of community-based participatory research (CBPR), will be a collaborative effort among the University of Minnesota, the Stair step Foundation (which has a coalition of 32 African American churches in the Twin Cities), and the Minnesota International Health Volunteers (MIHV).

Approach: The approach is multi-faceted and will engage indigenous communities including their health opinion leaders, health scholars and practitioners, and other "communities" of MUN researchers, clinicians, policy makers, and students. Through partnership and capacity building, they will plan to work collaboratively to create mutually beneficial and sustainable programs with the goal of producing knowledge that may be directly applied to improving community health. Along with the community partners, they plan to develop, implement, and evaluate innovative community health. They also will plan to develop, implement, and evaluate innovative community engagement and outreach programs with the ultimate goal of reducing health disparities. The approach is strong and appropriate.

Investigators: The qualifications and experience of the Core Director are appropriate.
Kolawole S. Okuyemi, M.D., M.P.H. - Director for the Program in Health Disparities Research and Associate Professor of Family Medicine at the University of Minnesota Medical School (1.80calendar months), will serve as Community Engagement and Outreach Core Director. Kathleen Thiede Call, Ph.D. - Associate Professor and DGS in the School of Public Health, Division of Health Policy and Management (0.60 calendar months), will serve as Co-Director of the Community Engagement and Outreach Core.

Environment: Excellent, the Medical School at UMN is the core of the state's largest medical complex. It has nationally recognized expertise in several key areas of research for the study of health disparities and community engagement.

Budget: Appropriate

CRITIQUE 3 OF THE COMMUNITY ENGAGEMENT/OUTREACH CORE

Significance: Community engagement effort that is planned and implemented by well trained investigators and with genuine input from the "community" is important to achieve meaningful outreach involvement in health disparities research. The CeHDRET Community Engagement and Outreach Core address this goal well.

Approach: The focus of the Community Engagement and Outreach Core is African Americans and African immigrants (Somali) in the Minneapolis areas. Community Collaborative Pilot Grants are planned and several community partners will be engaged.

Investigator: The Core Director – Kolawole S. Okuyemi, MD, MPH is highly qualified to lead the core. An Ilorin graduate, Dr. Okuyemi is the Director for Program in Health Disparities Research and Associate Professor of Family Medicine. Dr. Okuyemi has an impressive resume of research experience in minority populations, in mentoring trainees successfully and in obtaining extramural research funding. His publication record is outstanding. The Co-Investigators are well qualified. Ken Resnicow, the Consultant from the University of Michigan, School of Social Policy brings valuable expertise to the team.

Budget Recommendations: The budget is appropriate

Administrative Comments: *None*

CRITIQUE 4 OF THE COMMUNITY ENGAGEMENT/OUTREACH CORE

Significance: The Community Collaborative Planning Grants program is impressive; funded with institutional money, this program funded 5 grants in 2007 with at least one community co-investigator.

Approach: The plan to effectively subcontract $25,000/y x 5 years to the community partners (Stairstep and MIHV) is a high-risk but potentially very high-yield proposition. Money is power. This will represent true empowerment of the community organizations!

However, the community empowerment and substantive involvement goes beyond the money. For both projects, the investigators have carefully laid out plans to involve community organizations and community members in these activities that have been chosen based on specific community feedback. Very impressive!

Project 2 involves no apparent or anticipated connection with the community.

Investigator: Director, K. Okuyemi, is also Deputy Director of the administrative core; he is an African immigrant and has a strong record of research in smoking cessation among African Americans. Co-director Kathleen Call has extensive research in health disparities research and community health. Both are experienced researchers. Together they bring a strong experience in community-based research and are well qualified to lead the core.

Budget Recommendations: Budget is appropriate. The total budget for the CEOC is $946,000 over 5 years. It is refreshing to see this level of funds committed to this serious pair of projects. In

particular, the concept of giving the community organizations discretion and liberty to use the funds as they see fit to achieve the aims is compelling.

Administrative Comments: None

1D. RESEARCH/TRAINING AND EDUCATION CORE (SCORE DESCRIPTOR: EXCELLENT)

CORE LEADER (S): Vickers, Selwyn

DESCRIPTION (As provided by applicant): One of the current challenges in reducing health disparities and improving minority health is the lack of an adequate number of investigators, health care providers, role models, and educators. The deficiency in the number of these leaders compounds the problem of persistent health disparities. Thus the overall goal of the Research Training and Education Core will be to cultivate, train, and grow the pool of minority academic investigators. These goals will be developed in the context of the comprehensive goal of the Center for Health Disparities Research, Engagement, and Training. Critical to addressing these problems will be intramural and extramural partnerships. Thus we will build on existing programs within the University of Minnesota Medical School and Academic Health Center. Extramurally we will include partnerships with the traditional historically black medical institutions, specifically Howard University, Meharry Medical College, and Morehouse School of Medicine. The Research Training and Education Core has four specific aims: 1. Develop a new partnership with the Minnesota Future Doctors Program to select four of their highly talented students to engage in a pre-M.D./Ph.D. research training experience; 2. Develop a new partnership with the Office of Clinical Research Summer Pre-Doctoral Fellowship Program in Clinical and Translational Research to include highly talented health profession students from Howard University, Meharry Medical College, and Morehouse School of Medicine for a summer pre-doctoral health disparities research training experience; 3. Develop a cultural competency curriculum for medical students at the University of Minnesota Medical School; 4. Provide qualified investigators with the experience of preparing grant applications. We believe these programs will provide a critical opportunity to expand the pipeline for students who are from underrepresented minority backgrounds and for those working in health disparities and minority health. At the end of five years, we will have trained 20 undergraduate students in the MN Future Doctors program and 20 health science students from historically black universities.

CRITIQUE 1 OF THE RESEARCH/TRAINING AND EDUCATION CORE

Significance: The research training and education core will focus on two formal training programs targeting promising minority students interested in research at the undergraduate and medical school levels. These students will be given paid research positions with one of a group of 20 core mentors.

It is not specified whether the research projects will involve minority health or health disparities research, though many/most of the core mentors have research in this area.

Minority status does not appear to be a requirement for selection; though based on the pool of potential applicants, most will either be minorities or “rural, economically-challenged, or first generation”.

Approach: Two formal research training programs: undergraduate and medical student.

Common elements include a didactic curriculum (once weekly x 8-10 weeks) and a research project. Research project will be supervised by one of 20 core faculty mentors, listed in Table 1.

Undergrad program: One calendar year; 4 students chosen from the well-established Minnesota Future Doctors program (pool of 25 pre-med minority students who live in the Twin Cities area). Appears to be summer prior to junior year (immediately following the 6-week MFD Year 2 at Mayo clinic) with 20 h/w during Junior year (paid). Formal application and selection process, interviews.

Concerns: There does not appear to be a formal process for developing/supervising student's projects. How will adequate mentorship/oversight of students by mentors be ensured? Is there a process in place for review/approval of the project itself?

Pre-doctoral program: 10 weeks/one summer, for students in health professional schools. Partnering with OCR summer fellowship program/UMN, which has trained 30 scholars/3 years. Adding 4 students a year from 3 HBCUs. How will they ensure a meaningful research experience in such a short time period?

Concerns: The three letters of support from Howard (Dentistry, Medicine, and Pharmacy) are not on Howard university letterhead and are unsigned. The wording of all the letters is extremely similar, suggesting they have been written by the investigators, not the partners.

Concerns which apply to both programs: Neither of the Project PIs (Dr. Thomas, Dr. Israni) are listed as one of the core faculty members who will be available as mentors (p 344).

Cultural competency training for medical students: The investigators propose that they will participate in an overall redesign of the medical student curriculum which is planned for 2010 to ensure cultural competency is woven throughout the medical education program. The application is vague about how this will be done, and by whom. It sounds as if planning for this new curriculum is already well underway, and it does not appear that the core directors have been part of the curriculum planning that has occurred up to this point. It is unknown whether the investigators in this core were part of the MED 2010 "Disparities in Health Working group" in Appendix D.

Investigator: Dr. Vickers (director) and Dr Ahluwalia (co-director) – 2.4 months combined effort. They appear experienced and qualified as mentors. Also 1.8 mos administrator time.

Budget Recommendations: As requested

Administrative Comments: The three letters of support from Howard (Dentistry, Medicine, and Pharmacy) are not on Howard university letterhead and are unsigned. The wording of all the letters is extremely similar, suggesting they have been written by the investigators, not the partners.

CRITIQUE 2 OF THE RESEARCH/TRAINING AND EDUCATION CORE

Significance: Minorities are underrepresented in clinical medicine, clinical research, and education. Yet they are needed to create a culturally sensitive environment and provide role models for future generations. The Core's focus is to increase their numbers as clinicians, researchers, educators, and thus role models.

Approach: This core will 1) expand the partnership with the Minnesota Future Doctors Program (which enrolls 25 students annually) to select 4 talented students to engage in pre-MD/PhD research training, 2) Expand the office of Clinical Research Summer Pre-Doctoral Fellowship Program in Clinical and Translational Research to include talented health profession students from Howard University, Meharry Medical College, and Morehouse School of Medicine for summer pre-doctoral health disparities training, 3) Develop a cultural competency curriculum for medical students, students, faculty, and staff at the University of Minnesota, and 4) provide grant writing experience.

For the research training programs (undergraduate and health-related graduate students) there is cultural competence training (detail about the scope of this training was provided), a core curriculum, career development seminar series, and mentored research project. Detail about selecting mentors, guiding students, and evaluating their performance is provided. Routine evaluation of students and the

research training program will be conducted. Excellent detail about the availability and selection of trainees was provided.

Investigator: The Director of this Core is the Jay Phillips Professor and Chair, Dept of Surgery, Associate Director of Translational Research, and a senior scientist at the Masonic Cancer Center. He was a founding member of the National Center on Minority Health and Health Disparities. The Co-Director is the PI for the Center. Both are very well qualified. The Program Manager has relevant experience.

Budget recommendations: The budget appears to be commensurate with the proposed activities. (The amount for student travel to the Midwest conference seems excessive, but may foster attendance). Perhaps should be decreased.

CRITIQUE 3 OF THE RESEARCH/TRAINING AND EDUCATION CORE

Significance: Research training and education targeting minority investigators and trainees is critical to the success of health disparities research program. The CeHDRET research and education training core will an important role towards this objective.

Approach: The core lists four specific aims. Several innovative training programs are proposed. The core reaches out Howard University, Meharry Medical College and the Morehouse School of Medicine. The core functions are well integrated into the CeHDRET.

Investigator: The Principal Investigator – Selwyn M Vickers, MD, Jay Philips Professor and Chair, Department of Surgery is well qualified to lead this core. She was the PI of the University of Alabama NCMHD P60 grant before joining the University of Minnesota in 2006. Co-Investigators are well qualified.

Budget Recommendations: The budget is appropriate to the proposed task.

CRITIQUE 4 OF THE RESEARCH/TRAINING AND EDUCATION CORE

Significance: This core will help in reducing health disparities and improving minority health by creating adequate number of investigators, health care providers, role models, and educators. The deficiency in the number of these leaders compounds the problem of persistent health disparities. Thus the overall goal of the Research Training and Education Core will be to cultivate, train, and grow the pool of minority academic investigators. These goals will be developed in the context of the comprehensive goal of the Center for Health Disparities Research, Engagement, and Training. Critical to addressing these problems will be intramural and extramural partnerships. This will build on existing programs within the University of Minnesota Medical School and Academic Health Center and will include partnerships with the traditional historically black medical institutions, specifically Howard University, Meharry Medical College, and Morehouse School of Medicine.

Approach: The proposed core will benefit from the unique features of the partnering institutions and environment. The approach is acceptable and sound. The Research Training and Education Core will: 1. Develop a new partnership with the Minnesota Future Doctors Program to select four of their highly talented students to engage in a pre-M.D./Ph.D. research training experience; 2. Develop a new partnership with the Office of Clinical Research Summer Pre-Doctoral Fellowship Program in Clinical and Translational Research to include highly talented health profession students from Howard University, Meharry Medical College, and Morehouse School of Medicine for a summer pre-doctoral health disparities research training experience; 3. Develop a cultural competency curriculum for medical students at the University of Minnesota Medical School; 4. Provide qualified investigators with the experience of preparing grant applications. These programs may provide a critical opportunity to

expand the pipeline for students who are from underrepresented minority backgrounds and for those working in health disparities and minority health.

Investigators: The investigators are qualified to perform all of the activities proposed for this core. Core Director' Selwyn M. Vickers, M.D., is the Associate Director of Translational Research and a senior scientist in the Masonic Cancer Center. He is also the Jay Phillips Professor and Chairman of the UMN Department of Surgery, overseeing the administration of its academic, clinical and research missions. Dr. Vickers currently serves on the NIH SPORE Study Section. Dr. Vickers was a founding member of the Advisory Committee for the National Center on Minority Health and Health Disparities (NCMHD). He has published more than 100peer-reviewed manuscripts and is also PI of the new UMN Surgical Oncology Research Training Program (1T32 CA132715). Before coming to the University of Minnesota, Dr. Vickers served as the John H. Blue Professor of Surgery and as Chief of the Section of Gastrointestinal Surgery at the University of Alabama at Birmingham (UAB) from 2002 to 2006. While at UAB, he led the development of a pancreatic cancer research program, and served as the PI of the UAB Pancreatic Cancer SPORE (P20 CA101955) from 2003-2006, and currently serves as the Co-Pi of the UAB/UMN Pancreatic Cancer SPORE. In addition to his important work in translational cancer research, Dr. Vickers has been a significant leader in the minority health and health disparities field. While on faculty at UAB, with Drs. Mona Fouad and Edward Partridge, Dr. Vickers served as PI of the NCMHD grant (P60 MD00502) and Director of the Research Training Core prior to relocating to UMN. After moving to Minneapolis in 2006, Dr. Vickers has remained involved in the program as a consultant. As Director of this Core, Dr. Vickers will oversee the activities and personnel in this application, represent the Core during recruitment events, and conduct evaluations of all the students.

Environment: The environment is excellent. The UM has a long-time, excellent reputation with minority communities of African Americans.

Training in Responsible Conduct for Research: Appropriate

Budget: Appropriate

1E. RESEARCH CORE (SCORE DESCIPTOR: OUTSTANDING)

CORE LEADER (S): EVERSON-ROSE, SUSAN A

DESCRIPTION: (As provided by applicant) The Research Core for the proposed Center for Health Disparities Research, Engagement, and Teaching (CeHDRET) has two primary objectives. Our first objective is to promote research focused on health disparities and minority health at the University of Minnesota and with our Minneapolis and St. Paul metro area partners. The second objective is to provide an integrative "home base" for this research. Our goals are to improve the health of minority groups, and, ultimately, to eliminate health disparities across racial/ethnic groups. The Research Core has four Specific Aims. Aim 1 is to oversee the two primary research projects proposed for our Center. Project 1 is an innovative intervention study utilizing Community Health Workers and biomarker feedback to reduce African American children's exposure to secondhand tobacco smoke in the home and to encourage home smoking restrictions. Project 2 will use available national Medicare data to address disparities related to race/ethnicity and access to quality care in the management of and morbidity and mortality outcomes related to chronic kidney disease, an important, though often under-recognized public health problem disproportionately affecting African Americans. Aim 2 is to facilitate the development of innovative new research projects for external funding that address minority health concerns and health disparities. Aim 3 is to develop a collaborative and integrative approach for health disparities and minority health research. Aim 4 is to disseminate research findings to the academic community, community groups, health care providers and policy makers. We will achieve these aims by providing research infrastructure support; offering monthly "work in progress" research seminars to share work at various stages and facilitate exchange of ideas; establishing a Research Review Committee that provides a scientific "mock review" to maximize success of grant applications; and sponsor an annual "Accelerating the Future" research symposium highlighting

research of our investigators and trainees, featuring a keynote national speaker, and giving mentor, community leadership, and investigator awards. By integrating resources, interests, and opportunities related to health disparities and minority health, we will build research capacity and develop cutting edge research projects and programs.

CRITIQUE 1 OF THE RESEARCH CORE

Approach: The two primary projects do not appear to be thematically connected at all.
The activities of the research core are well-described and specific, and laid-out in aims (p220). Infrastructure support is described. Particularly impressive are their plans to

- encourage collaboration of research across the University (through annual research symposium, quarterly luncheon series);
- to establish a research review committee which will offer mock reviews, advise new investigators
- to foster new research projects via a University-funded pilot grant program. Up to 4 awards, $50,000 each, per year beginning year 2… with a system of formal review.

The process for selection of the two primary projects is well-described: an internal RFA soliciting a one-page proposal. 10 applications (8 listed in table 1?) were reviewed by a committee. Oversight of projects well-described, and will occur via biannual progress reports, annual meeting with executive committee.

Investigator: Director Susan Everson-Rose: social epidemiologist and psychopysiologist; Co-director Mary Story: Epidemiologist. Both directors are experienced investigators with established track records of managing large research projects. Both have the experience and commitment to fulfill their obligations. Plans for communication and cooperation are well-delineated.

Environment: Research environment is strong and supportive. Plans to use institutional funds for various aspects of the proposed Center indicate a firm institutional commitment to the project.

Additional Review Criteria:

Human Subjects: Applicable

Risks and Protections: Acceptable

Inclusion of Women: Acceptable;

Inclusion of Minorities: Acceptable

Inclusion of Children: Acceptable

Targeted Recruitment for Clinical Trials: Acceptable

Vertebrate Animals: Not Applicable

Budget Recommendations: As requested

Administrative Comments: None

CRITIQUE 2 OF THE RESEARCH CORE

Approach: This Core aims to promote research focused on health disparities and minority health with University and metro area partners and to provide an integrative hub for this work. There are 4 aims: 1)

to oversee 2 independent research projects, 2) facilitate the development of new research to address this domain of minority health and health disparities, 3) develop and nurture collaborative, integrative, and translational research models in this domain, and 4) disseminate research findings broadly to scientists and the community. A broad solicitation across the academy for potential applicants was conducted, and two projects have been selected that addresses gaps among discovery, development, and delivery of health-related factors. One project focuses on improving minority health through smoking cessation and the other focuses on health disparities in chronic kidney patients.

For Aim 2 the Core is developing a Research Review Committee to provide feedback on grant applications prior to submission (it is composed of the Core Directors and Co-Directors, and 3-5 selected ad hoc reviewers from the University), to ensure the proposals are culturally sensitive, to have access to the resources at UM Centers (e.g., Health Survey Research and Biostatistics, Design, and Analysis Centers), and oversee the HD Pilot Research Grants Initiative (funded by the UMMS).

For Aim 3 the Core is sponsoring Accelerating the Future – a day-long University wide Research Symposium for investigators, policy makers, clinicians, and community partners, coordinating monthly trans-disciplinary research seminars, hosting a quarterly, ‘building bridges’ luncheon series to foster collaborations across the university (laudable and truly aiming to be trans-disciplinary), and coordinate the Community Dialogue Series *research* meetings quarterly.

This Core will fund the Accelerating the Future Symposium Speaker Honorarium (for the keynote) and an External Grant Reviewer Honorarium (the Core’s Research Review Committee will hold a mock NIH-like grant review, with those receiving scores of excellent given the possibility of an external review by a consultant).

Investigator: The Director of this Core is the Associate Director for the Program in Health Disparities Research. She has appropriate research and administrative experience to oversee this Core. The Co-Director is Professor of Epidemiology and Community Health and Professor of Pediatrics. She has served as Associate Dean for student and academic affairs in the School of Public Health and is presently the Director of the National Program Office of the Robert Wood Johnson Foundation-funded Healthy Eating Research Program. The Program Manager has extensive experience with University and community initiatives, and is PM for another Core***.

Environment: The physical resources, leadership, and university and community partnerships are excellent to foster the aims of this Core.

Human Subjects: Applicable

Risks and Protections: Acceptable

Inclusion of women, minorities, children: A G1, M1,C3 A(both, both, adults)

Targeted Recruitment for clinical trials: Acceptable

Vertebrate Animals: NA

Animal Care and Use: NA

Budget Recommendations: The budget appears to be commensurate with the proposed activities.

CRITIQUE 3 OF THE RESEARCH CORE

Significance: The CeHDRET research core will harmonize the investigative activities of the Center. This is an essential task in helping the Center to achieve its goals.

Approach: Two objectives are listed for the Research Core. Several functions are outlined. The core will play important roles in the two specific research projects as it has already done in the process of selecting the projects prior to submission of the grant proposal.

Investigators: Dr. Everson-Rose is well qualified to direct the Research Core. The co-Investigators are well qualified.

Environment: The environment at the University of Minnesota is outstanding in every respect necessary to accomplish the missions of the CeHDRET

Budget Recommendations: The budget is appropriate for the proposed tasks.

Human Subjects: Applicable (G1, M2, C1)

Risks and Protections: Acceptable

Inclusion of Women: Acceptable: Code=1

Inclusion of Minorities: Acceptable/ Code=2

Inclusion of Children: Acceptable/ Code=1

Targeted Recruitment for Clinical Trials: Acceptable

Vertebrate Animals: Not Applicable

Animal Care and Use: Not Applicable

CRITIQUE 4 OF THE RESEARCH CORE

Approach: This Research Core aims to promote research focused on health disparities and minority health at the University of Minnesota and to provide an integrative "home base" for research. The Research Core will oversee two primary research projects: the first intervention study utilizing Community Health Workers and biomarker feedback to reduce African American children's exposure to secondhand tobacco smoke in the home and to encourage home smoking restrictions. The second project will use available national Medicare data to address disparities related to race/ethnicity and access to quality care in the management of and morbidity and mortality outcomes related to chronic kidney disease, an important, though often under-recognized public health problem disproportionately affecting African Americans. The core will also facilitate the development of innovative new research projects for external funding that address minority health concerns and health disparities. It will develop a collaborative and integrative approach for health disparities and minority health research and disseminate research findings.

Approach: The core will provide research infrastructure support; offering monthly "work in progress" research seminars to share work at various stages and facilitate exchange of ideas; establishing a Research Review Committee that provides a scientific "mock review" to maximize success of grant applications; and sponsor an annual "Accelerating the Future" research symposium highlighting research of investigators and trainees, featuring a keynote national speaker, and giving mentor, community leadership, and investigator awards. By integrating resources, interests, and opportunities related to health disparities and minority health, will build research capacity and develop cutting edge research projects and programs. This approach is well sound and feasible.

Investigators: The Research Core Director will be Susan A. Everson-Rose, Ph.D., M.P.H., Associate Professor in the Department of Medicine and Associate Director for the Program in Health Disparities Research at the University of Minnesota Medical School. Dr. Everson-Rose is a social epidemiologist and psychophysiologist who has considerable expertise and has published widely on the role of stress, psychosocial factors and socioeconomic factors in the development and progression of cardiovascular diseases, stroke and diabetes. She presently is funded by NHLBI to examine how individual and neighborhood level indicators of psychosocial stress contribute to stroke risk in an elderly population of African-Americans and Whites residing in Chicago and whether psychosocial vulnerability contributes to racial disparities in stroke. The Research Core Co-Director will be Mary Story, Ph.D., Professor in the School of Public Health and the Medical School's Department of Pediatrics with extensive experience in research and administration. Dr. Story served as Associate Dean for student and academic affairs in the School of Public Health until 2005, at which time she became Director of the National Program Office of the Robert Wood Johnson Foundation-funded Healthy Eating Research program. She currently is PI on Bright Start (R01 HL078846), a NIH-funded school and family-based obesity prevention study on the Pine Ridge (American Indian) reservation with kindergarten and first grade children. She has authored 280 scientific journal articles and been the PI on more than $15million in NIH funding, and the Co-Pi and Co-I on more than $20 million in NIH funding. Dr. Story has received several awards for her community-based work and research; in 2007 she was inducted into the University of Minnesota Academic Health Center (AHC) Academy for Excellence in Health Research, the high recognition of excellence in AHC faculty research.

Environment: The environment is well suited for the project. The application details several plans for resource allocation, coordination and utilization. Strong letters of support are included.

1F. The RESEARCH PROJECTS WITHIN THE RESEARCH CORE:

1F-1. Research PROJECT 1 - Community Outreach and Biomarker Feedback for Smoke-Free African American Homes (PRIORITY SCORE: 184)

PROJECT LEADER (PD/PI): THOMAS, JANET L

DESCRIPRION (Provided by the Applicant): Exposure to environmental tobacco smoke (ETS) early in life increases the risk of sudden infant death syndrome, asthma, and respiratory infections contributing to more than 5,000 premature deaths among children in the US each year. African American children suffer disproportionately from the consequences of ETS exposure with well documented higher rates of sudden infant death and asthma. While the roots for these disparities are complex (e.g., poverty, poor housing conditions, environmental allergens), exposure to ETS is a prominent and quickly reversible cause of excess morbidity and mortality. We propose to address this deficit by providing culturally-sensitive biomarker feedback to the smoking parent/caregiver on their child's exposure to tobacco toxins. We will use a client centered motivationally enhanced counseling style to deliver the intervention. Further, delivery of this intervention by community health workers (CHWs) will further increase the salience of the proposed biomarker feedback intervention. With these points in mind, we propose a two-arm randomized clinical trail (N=180) to determine the efficacy of culturally sensitive biomarker feedback documenting a child's exposure to tobacco toxins (NNAL, nicotine, cotinine) to reduce home ETS exposure among African American children. Participants (i.e., the smoking parent or caregiver) will be randomized to receive one of two treatments: Tx1: General Home Safety Intervention - Contact Control Group (Lead Biomarker Feedback) Tx2: Home ETS Reduction Intervention - Treatment Group (Tobacco-Specific Biomarker Feedback)
We hypothesize that tobacco-specific biomarker feedback will result in decreased home ETS (air nicotine levels) through increased parental smoking cessation and/or adoption of complete home smoking bans. This study will identify an innovative and effective strategy for reducing ETS exposure among African American children. The proposed delivery of culturally sensitive biomarker feedback can

serve as a template for future efforts to bring the promise of personalized medicine to underserved populations.

CRITIQUE 1 OF THE RESEARCH PROJECT 1

Significance: Exposure to environmental tobacco smoke (ETS) increases the risk of many health risks for children, with African American children having a much greater burden than other groups. This project aims to provide child biomarker feedback to smoking adult parents/caregivers.

Approach: A 2-arm randomized trial is proposed (N=180) to determine the efficacy of culturally sensitive biomarker feedback to parents/caregivers about child's exposure to ETS. The control group receives Lead biomarker feedback, and the treatment group receives tobacco-specific biomarker feedback. The expectation is that exposure to ETS feedback will reduce in-home air nicotine levels (primary outcome) and biomarkers in children (urinary cotinine; secondary outcome) over time because parent/caregivers have either reduced their smoking rates or have reduced smoking in the home (a mediator; and various psychological mediators are examined – e.g., optimistic bias, self-efficacy, social support). Community health workers will be trained to deliver the multi-session, home-based interventions (1 for smoking cessation, 1 for lead) using motivational counseling, this feedback will happen only once during week 2. There are clear inclusion/exclusion criteria.

The biggest concern with this intervention is that there are numerous approaches to motivate smoking cessation in smokers. Use of a standard of care in smoking cessation, rather than a lead-based comparison, would be a stronger test for hypotheses? The proposed intervention, while controlling for time and attention, is a very weak test for this intervention, which is based in part on motivational interviewing. A stronger test for the intervention would do much more for bringing 'science' to the 'community.' The use of the 7-day air badges as a primary outcome is somewhat worrisome because there is no way to ensure they were utilized the entire time.

Innovation: ETS biomarker feedback may be an effective strategy to reduce AA parental/caregiver tobacco use in the home. There is a paucity of research in this population that has a larger ETS burden and attention to constructs important for behavior change according to self-determination theory (i.e., autonomy, competence, and relatedness) have not been tried and should increase success.

Give control group no biomarker feedback and no motivational counseling.

Investigators: The PI and Co-Investigators have a track record of research and funding in tobacco cessation research, and with culturally relevant experience. There are also Co-Investigators with biomarker collection, analysis, and feedback experience. Two community health workers will be hired from the community and trained in delivering the intervention. Two external consultants, one with expertise in smoking cessation and interventions, and one with expertise in the effects of airborne materials on health, will be utilized. Two internal consultants (no funds requested) are also delineated.

Environment: The resources to conduct this research are very good. The community partnership and subcontract for ensuring a good response rate and for obtaining community health workers is very good.

Additional Review Criteria:

Human Subjects: A; DSMP in place, Certificate of Confidentiality will be sought What precautions will be made to ensure children will not be coerced into participating from their parent/caregiver, or that children will not be punished by caregiver for implicating him/her in 'bad' behavior?

Risks and Protections: Acceptable

Inclusion of women, minorities, children: A, G1, M2, C1 (both, minority, both)

Targeted Recruitment for Clinical Trials: A

Vertebrate Animals: NA **Animal Care and Use:** NA

Budget recommendations: The budget seems commensurate with proposed activities.
To help ensure adequate response rates for outcome assessments a subcontract with a community health center is in place. Two Community Health workers will be hired to carry out all in-home intervention activities and participate in weekly research meetings.

CRITIQUE 2 OF THE RESEARCH PROJECT 1

Significance: This project addresses a critical need to eliminate child exposure to environmental tobacco smoke (ETS) in African American homes. African American children have higher levels of cotinine-verified ETS exposure than non-Hispanic whites and Mexican Americans. The central hypothesis is that enhancing the cultural relevance and messages alliance of a client-centered, motivational counseling intervention and providing feedback on tobacco-specific biomarker levels in children in the homes will lead to decreased ETS exposure in the children, adoption of home smoking restrictions, and more, accurate parental perceptions of health risks of smoking for their family. To test this hypothesis, the applicants propose a two-arm randomized, controlled trial investigating the efficacy of feedback on tobacco-specific biomarkers in children and a tailored; motivationally enhanced counseling approach to decrease home ETS exposure. The biomarker feedback and counseling will be delivered by African American Community Health Workers (CHWs) in home visits. The control arm of the trial will receive information about home safety in an equal number of home visits. Participants will be 180 African American smokers with at least one child in the home recruited from North Minneapolis and surrounding neighborhoods where the majority of African Americans in Minneapolis reside. Research staff will be housed in the Center's new space in the University's community-based building located in the Northside Minneapolis community (see description in Administrative Core). This project builds upon Dr. Thomas' prior and currently funded work developing novel smoking cessation interventions for minority populations, including use of partner support and biomarker feedback, and extends this program of research to maximizing the cultural relevance of the intervention. This study has the potential to contribute to the field of evidence-based medicine. It may also help in reducing or eliminating health disparity among African American.

Approach: The conceptual, design, methods, and analyses adequately developed, well integrated to the aims of the project. The objective in the proposed application is to test whether the delivery of biomarker feedback documenting a child's exposure to environmental tobacco smoke will increase smoker quit rates and/or, increase the adoption of home smoking bans, ultimately decreasing home ETS exposure. They will train community health workers to use a motivationally enhanced counseling strategy to deliver the biomarker feedback intervention to increase message salience and enhance motivation. The proposed delivery of culturally sensitive biomarker feedback can serve as a template for future efforts to bring the promise of personalized medicine to underserved populations. However, the approach is not very innovative.

Investigators: This team is well-qualified to carry out the proposed study. This is a multidisciplinary clinical team that comprises a general internist with expertise in health disparities and diabetes Janet L. Thomas, Ph.D. - Assistant Professor of Medicine, is Project Leader for Project 1. Dr. Thomas is Assistant Professor in the Division of General Internal Medicine (Department of Medicine) at the University of Minnesota (UMN), and a member of the University of Minnesota Cancer Center. She is a licensed clinical psychologist and former tobacco research fellow at the Mayo Clinic, Nicotine Dependence Center, training under the guidance of Drs. Richard Hurt and Christi Patten. Dr. Thomas is uniquely suited to serve as the Principal Investigator for this proposal based upon her prior experience on multiple tobacco control projects focusing on behavioral interventions with African American

smokers. These projects include behavioral and pharmacologic interventions, qualitative analyses, and survey development. For this project, Dr. Thomas will provide overall study direction and will work closely with the team from North point Health and Wellness Center, Inc. (subcontract) to supervise the implementation of the CHW delivered motivationally-enhanced delivery of the biomarker feedback and home smoking intervention. Dr. Thomas will be responsible for the development of the assessment surveys and will work closely with Drs. An, Hennrikus and Borrelli in developing the counseling intervention. She will train the study research staffon participant enrollment, informed consent, assessment completion, and will work with Dr. Ramirez-Barrett (North point Health and Wellness Center, Inc., Director) to monitor the fidelity of the CHW-delivered counseling intervention. She will work with Drs. Hennrikus and Borrelli to develop the counselor training materials for this project and will oversee the counselor training sessions. With the Project Coordinator, she will run weekly investigator meetings (with Dr. Ramirez-Barrett joining by phone).

Jasjit S. Ahluwalia, M.D., M.P.H., M.S. - Associate Dean for Clinical Research in the Medical Schooland Professor of Medicine and Professor of Epidemiology and Community Health , is Co-Project Leader of Project 1. Dr. Ahluwalia is a nationally recognized investigator with a track record of research funding and mentoring. Lawrence C. An, M.D., M.P.H. - Associate Professor of Medicine, will serve as Co-Investigator for Project 1. Dr. Larry an is an Associate Professor in the Division of General Internal Medicine (Department of Medicine) at the University of Minnesota. He is a general internist and former Robert Wood Johnson Clinical Scholar (University of Michigan). Dr. An is a seasoned tobacco researcher, having received two smoking intervention R01 grants from NHLBI to investigate web based access to NRT to facilitate smoking cessation and another designed to provide college students with behavioral strategies to quit via an internet site. Deborah Hennrikus, Ph.D. - Associate Professor in the School of Public Health, Division of Epidemiology and Community Health, will serve as Co-Investigator of Project 1. Dr. Hennrikus is a behavioral scientist and an Associate Professor in the Division of Epidemiology and Community Health, School of Public Health, University of Minnesota. She has extensive experience in community-based research projects to assess interventions to decrease to exposure to environmental tobacco smoke.

Environment: The investigative team has access to patients in these clinics and a letter of support signed by both the Executive Director and Medical Director is strong.

Protection of Human Subjects from Research Risks: Appropriate

Inclusion of Women Plan: Appropriate

Inclusion of Minorities Plan: Appropriate

Inclusion of Children Plan: Appropriate

Budget: Reasonable.

CRITIQUE 3 OF THE RESEARCH PROJECT 1

Significance: Environmental Tobacco Smoke (ETS) is an important cause of morbidity and mortality with disproportionate impact on minority populations. Proven interventions that could alleviate this exposure particularly in children are needed

Approach: Client centered motivationally enhanced counseling will be delivered to the smoking parent or caregiver in a randomized fashion to two treatment groups: Lead Biomarker Feedback and Tobacco-Specific Biomarker Feedback. Two hypotheses will be tested on the outcomes: air nicotine level (primary outcome) and smoking cessation or complete smoking bans.

Innovation: The experimental design is innovative

Investigators: The investigators are well qualified

Environment: The environments at the University of Minnesota and the Northwest Health and Wellness Center, Inc are adequate

Additional Review Criteria:

Human Subjects: Applicable (G1, M2, C1)

Risks and Protections: Acceptable

Inclusion of Women: Acceptable: Code=1

Inclusion of Minorities: Acceptable: Code=2

Inclusion of Children: Acceptable: Code=1

Targeted Recruitment for Clinical Trials: Acceptable

Vertebrate Animals: Not Applicable

Animal Care and Use: Not Applicable

Budget Recommendations:
Personnel budget effort appear to be excessive in several categories

- The budgeted effort for the PI is excessive and should be reduced. 20% is suggested
- The budgeted effort for each of the three co-investigators effort is excessive. 5% effort is suggested for Drs. Ahluwalia, An, Hennrik us and Luo
- The budgeted effort for Database Manager is excessive. 10% effort is suggested

CRITIQUE 4 OF THE RESEARCH PROJECT 1

Significance: ETS exposure in children is an important, modifiable public health concern – associated with asthma, SIDS, and possibly long-term risk for malignancy and CVD. ETS exposure is higher among AA children as compared with white children. But is there evidence that childhood diseases resulting from ETS are disparity conditions?

Aims 1 and 2 are closely related, and really should be considered sub-aims: two different ways of measuring the same thing. This home-based motivational-interviewing intervention seems quite labor-intensive. If successful, will it be practical to implement ? Who will pay for it?

Approach: Staged phases including focus group and pilot phase prior to RCT are impressive and seem to be well-designed and likely to succeed. The intervention will be developed in the initial phases with community feedback.

How will urine samples be collected on children that are not yet toilet trained?

Tobacco use is higher in African-American men as compared with African-American women; yet it is likely that most of the adults recruited into the study will be mothers rather than fathers. This is a potential weakness.

Innovation: Use of focus groups to pilot and refine the cultural relevance of the intervention is impressive and innovative. There is a thorough and systematic plan for analysis of focus group data.

The plan to assess psychological motivators for change (exploratory aim) is innovative and may be interesting.

Investigators: Application clearly demonstrates expertise and experience in conducting studies in smoking cessation using behavioral home-based interventions; also experienced in quantifying ETS exposure.

Environment: Adequate/supportive

Human Subjects: Though all participants will receive general information on smoking cessation, it does not appear that the control group will receive even basic educational information on the harmful effects of ETS exposure in their children. This seems negligent. HS protection for the kids is not clearly mentioned.

Risks and Protections: Acceptable

Inclusion of Women: Acceptable. They project 50/50 men and women; but more women are likely expected than men… Table 6 is not likely to accurately reflect the gender distribution.

Inclusion of Minorities: Acceptable G2A

Inclusion of Children: Acceptable C1A

Targeted Recruitment for Clinical Trials: Acceptable

Vertebrate Animals: Not Applicable

Budget Recommendations: As requested

Administrative Comments: None

1F-2 RESEARCHG PROJECT 2 - Health Disparities in Treatment, Cardiovascular Morbidity and Mortality of Chronic Kidney patients in the US (PRIORITY SCORE 269)

PROJECT LEADER: ISRANI, AJAY K

DESCRIPTION (as provided by applicant): Chronic kidney disease (CKD) affects an estimated 26 million Americans (or 13% of the adult U.S. population), and it is well known that CKD is an independent risk factor for atherosclerotic heart disease (ASHD) and mortality. These complications of CKD are more common in African Americans than Whites. These complications can be prevented by timely referral to a nephrologist and quality CKD care in order to prevent ASHD. Our preliminary studies show regional variations in lack of appropriate physician follow-up. Disparities in delivery of quality CKD care and their impact on the worse outcomes seen in African Americans have not been adequately described for Medicare beneficiaries with CKD. Therefore, it is important to determine whether physicians are adhering to existing evidence based guidelines which call for recognizing CKD as a potent risk factor for cardiovascular disease and aggressively monitoring serum creatinine, lipids, calcium-phosphorus, hemoglobin A1c (in diabetics) and parathyroid hormone. Other aspects of quality CKD care for African Americans entail providing preventive healthcare such as influenza vaccinations annually and timely referral to a nephrologist.
The specific aims are: 1) To determine if African Americans are less likely than Whites to receive quality CKD care. (2) To determine if quality of CKD care independently explains the worse outcomes in African American Medicare patients. Outcome measures .include patient death and atherosclerotic heart disease

(ASHD) events. (3) To determine if health service areas in the US with a disparity in quality CKD care are associated with a low density of providers (nephrologists and primary care physicians), the ratio of primary care physicians to nephrologists compared to HSAs with no disparity. This research project will use the national Medicare 5% sample, which represents all claims from a random subset of 8 million patients on Medicare from 1999-2006, making this the largest CKD study to the best of our knowledge. Our study will also validate an algorithm to determine CKD using Medicare claims data, which could be used with other administrative datasets in the future to examine whether patients are receiving quality CKD care. It will then compare quality of CKD care in African Americans vs. Whites and determine the HSAs with disparities. In the future, using claims data to study process of care characteristics in chronic diseases is an exciting area that can have implications for disparities research well beyond CKD.

CRITIQUE 1 OF THE RESEARCH PROJECT 2

Significance: Chronic kidney disease (CKD), its treatment and outcomes is most emblematic of health disparities in the U.S. This project could potentially identify important underlying factors responsible for the disparities in CKD

Approach: The project is a retrospective cohort analysis of the Medicare 5% sample which includes approximately 240,000 subjects with CKD from 1999 to 2006. Three specific aims are proposed to: determine if AA are less likely to receive CKD care; determine if quality of CKD care explains worse outcomes in AA; and determine whether Health Services Areas (HSAs) with disparity in the use of CKD preventative measures have low density of providers (nephrologists and primary care physicians). In addition, the Medicare claim data that will be used for the study will be validated by examining 1,170 cases in the NHANES national data from 1988-1994.

This research project is not integrated into the Research Core of the Center.

Major strengths
- Qualification of the investigative team
- The study would be the largest CKD cohort ever studied to explain racial disparities in CVD and outcomes among subjects with CKD

Weaknesses
- The study is limited in its application to subjects aged 65-95
- The validation data set is not contemporaneous with the 5% Medicare sample (1999 to 2006) that is being studied
- Using claims data to define CKD even when based on an algorithm that has been extensively used by the study group for 6-7 years is fraught with inaccuracies.

Innovation: A secondary analysis for specific aim 2 will use structural equation modeling to determine weather preventative measures *moderate* the outcomes of atherosclerotic heart disease and death in AA. This is a novel application of this technique to the CKD population.

Investigators: The Project Leader - Dr. Israni is an Assistant Professor of Medicine and nephrologist at the University of Minnesota. He has significant experience in working with large datasets including the USRDS. He is supported by an outstanding group of senior investigators including a renowned, pioneering transplant nephrologist and clinician scientist (Bert Kasiske).

Environment: The environment at the Minneapolis Medical Research Foundation is outstanding.

Additional Review Criteria:

Human Subjects: Applicable (G1, M2, C3)

Risks and Protections: Acceptable

Inclusion of Women: Acceptable: Code=1

Inclusion of Minorities: Acceptable: Code=2

Inclusion of Children: Acceptable: Code=3

Targeted Recruitment for Clinical Trials: NA

Vertebrate Animals: Not Applicable

Animal Care and Use: Not Applicable

Budget Recommendations:
- The duration of the project is excessive. A two-year timeline and budget is suggested.
- 22,000 to more than 40,000 per year (55% of salary) in computer services is outrageously excessive. This should be reduced to $10,000 per year for each of two years.

CRITIQUE 2 OF THE RESEARCH PROJECT 2

Significance: CKD is an important disparity condition: the incidence of ESRD is significantly higher in African Americans than whites. The author's hypothesis – that disparities in CVD and mortality outcomes associated with CKD may be attributable to differences in access to quality CKD care – seems reasonable and important. They will also test one hypothesis regarding the underlying forces which result in disparities in quality of care (low density of providers and ratio of PCP to nephrologists).

They discuss how the results of their research will inform future CKD screening/detection programs such as the KEEP program, but given the approach (studying only patients with medicare dx of CKD) its unclear what the relevance will be for screening/detection.

Approach: There are major flaws in their approach; some arising by the nature of the study design (i.e, a retrospective cohort selected based on Medicare claims) and some due to specific design decisions made by the investigators. The most important flaws are

1. the choice of ICD-9 codes that will define their sample;
2. the choice of "quality of care" measures being inappropriate for their target population; and
3. the abbreviated window for assessment of primary health outcomes (ASHD/mortality).

The investigators do not define their target population or "CKD". It is implied that they will study CKD stage 1-4, but this is not made explicit. The "quality of care" indicators that they will measure are not appropriate for CKD 1-2, but for CKD 3-4 only.

Use of Medicare claims (5% medicare claims dataset) to identify their study subjects is problematic for several reasons.

1. Population studied is limited to Medicare beneficiaries. Patients <65yo will not be studied; the uninsured will not be studied. The authors cite lack of insurance as a possible reason for disparities in CKD care; this cannot be ascertained with their study design.
2. Incomplete coding. CKD which is unrecognized/unacknowledged by a primary caregiver (or in cases where the patient is not receiving regular care) will be missed. The authors cite poor recognition/acknowledgement of CKD by primary providers as a possible source of disparity. All these patients will be missed.

3. “Definition” of CKD: (p. 309-310). Choice of specific ICD-9 codes for inclusion is far too broad, and will include large numbers of subjects without CKD: 9 of the 38 ICD-9 codes used for inclusion are not characterized by CKD, and several others are questionable. Since only one inpatient claim is required for inclusion, a large number of patients without CKD will be included. It is as if they took as broad a swathe as possible, including any diagnosis which has the word “kidney” or “renal” in it to maximize their sample size. This definition does not match up with the quality of care outcome measures chosen (see below).
4. “At least one inpatient OR at least two outpatient claim codes” (p. 309-310); “at least one inpatient AND at least two outpatient claim codes” (p. 314). This is a critical distinction. Both have flaws (the latter: patients will only be included if they are hospitalized during the study period).
5. “Our CKD claims algorithm will be validated and fine-tuned as part of this proposal” – This should be a major aim, not just a two-paragraph side note. The authors need to describe their validation procedure in more detail.
   a. How do they define CKD (ie, what is their gold standard)? – NKF stages themselves are designed for coding/billing purposes and are not rigorous enough to stand alone as research definitions.
   b. They propose to validate their claims strategy in 1170 subjects *with* CKD, ie, sensitivity; will they also assess false positives/specificity, which I see as a more important weakness? i.e, will they determine how often patients without CKD are inappropriately characterized as having CKD?
   c. What is their goal/standard for specificity and sensitivity? Will specific diagnoses be discarded if they do not meet certain sense/spec cut-offs? What is the procedure for making this determination?
   d. Will this assessment be completed prior to, or contemporaneously with, completion of the proposed study? Timeline on p. 322 suggests it will be completed (and Aim 1 analysis will begin) in the second half of the first year. This does not seem realistic, and does not allow for time to validate/modify/revalidate their algorithm in a systematic way.
6. Investigators cite prior work using Medicare claims database to assess disparity outcomes; but this prior work (patterns of care for kidney transplant recipients; access to transplant) studies populations who have de facto automatic medicare eligibility; AND the diagnosis codes for these conditions are much clearer and more likely to be coded properly. Neither of these important conditions holds for the current study.

Outcome Measures

Their definition of “quality CKD care” is questionable given their chosen outcomes. Is yearly influenza vaccination likely to be associated with excess CVD and mortality outcomes?

Table 4 (p. 304) – They took K-DOQI guideline recommendations for CKD stage 3-4 and apply them as standard of care in a population which appears to include CKD 1-2 - and given the flaws in their design, will also include many patients without any CKD at all. Specifically, Ca/P/PTH once a year are recommended only for CKD stage 3+; nephrologist visit is explicitly recommended only for CKD stage 4+; more than yearly Cr is recommended only for CKD stage 3+.

In addition, they omit several measures that are considered to be important standards of care (some of which can be assessed using Medicare claims, many of which cannot); ie, measurement of albuminuria in diabetics; measurement and control of blood pressure; screening for anemia; timely dialysis access placement.

Late referral to a nephrologist among African Americans appears to already be well-established.

Worsening renal function, or at least progression to ESRD (which is easy to measure in their dataset), should be one of their outcomes.

Mortality and ASHD in YEAR 3 ONLY will be counted. Patients enrolled in year 1 who die in year 2 will be excluded; mortality and ASHD events in year 4+ will not be measured. This is severely limiting. At a minimum, mortality and ASHD events in year 2+ for which data is available should be included as endpoints.

For Aim 3 (looking at relationship between density and ratio of providers and disparities) – they propose to examine the association between provider density and disparities in preventative measures. They should also look directly for an association between provider density and disparities in *outcomes* (ASHD, mortality). For example, patients may be less likely to see a nephrologist yearly in an area with lower density of specialists; but this may or may not translate into worse outcomes.

Innovation: The authors have already shown in their preliminary data that, in the medicare claims dataset from mid-1990s, AAs are less likely to have lipid testing, influenza vaccination, PCP visit, and HbA1c testing (for diabetics). It seems they are measuring many of the same endpoints, just in a different subgroup (those with CKD).

Investigators: Dr. Israni's degree is given as Ph.D. on the face page (p 292) but as M.D., M.S. under personnel/budget justification. He is a nephrologist at Hennepin county Medical Center. He is a K23 awardee and project leader on a U19. He is experienced in studying health disparities using Medicare claims data (post-transplant care). Dr. Kasiske, his K23 mentor, is Co-investigator. He is also experienced user of Medicare claims dataset to study transplant access disparities.

Environment: Appears to be satisfactory.

OVERALL EVALUATION: Although disparities in CKD care and progression are an important cause of increased morbidity and mortality in African Americans in the United States, there are several critical flaws in the design of this proposal which will render its conclusions close to meaningless. Because of this, enthusiasm for this proposal is quite low and this reviewer rated it as barely acceptable.

Additional Review Criteria:

Human Subjects: Applicable

Risks and Protections: Acceptable

Inclusion of Women: Acceptable G1A

Inclusion of Minorities: Acceptable M2A

Inclusion of Children: Acceptable/ C3A

Targeted Recruitment for Clinical Trials: Acceptable/

Vertebrate Animals: Not Applicable

Budget Recommendations: As requested

CRITIQUE 3 OF THE RESEARCH PROJECT 2

Significance: This project addresses racial/ethnic disparities and access to quality care that impact the management and ultimately the morbidity and mortality associated with chronic kidney disease (CKD). Minorities, especially African Americans, experience a disproportionate burden of CKD, including faster progression to ESRD requiring dialysis or kidney transplantation. CKD contributes to

significant impairments in quality of life and is a potent risk factor for cardiovascular disease. Evidence suggests minorities are less likely than Whites to be aware of their CKD, raising the possibility that disparities in CKD outcomes may result from disparities in management and treatment of CKD risk factors in minority groups. The central hypothesis is that the excess morbidity and mortality outcomes related to CKD observed in African Americans result from disparities inequality of CKD care. A secondary hypothesis is that disparities in quality of CKD care vary within regions of the US. Dr. Israni and colleagues will use available Medicare Claim s data to determine quality of CKD care received by African American patients, compared with White patients, and whether poorer quality care contributes to excess morbidity and mortality related to CKD that has been observed in African Americans. These investigators also will determine which parts of the US have greater disparity in quality of CKD care and whether density of providers (primary care physicians and nephrologists) or ratio of primary care physicians to nephrologists contribute to disparities in CKD care. Using available national administrative data enables Dr. Israni and colleagues to test their hypotheses in a highly efficient, cost-effective manner. This project builds upon Dr. Israni's prior work, funded by the Robert Wood Johnson Foundation, examining disparities in minority access to nephrologists following kidney transplantation and addresses an important disparity in CKD outcomes.

Approach: the approach is sound and appropriate given the study goals and objectives. This study is very important, unique in its focus. A major strength of this project is the experience of the investigative team. The preliminary studies clearly demonstrate the ability of the team in conducting the project. This study will describe the quality of care for African American patients with chronic kidney disease in the United States. It will determine if African Americans are less likely than Caucasians to receive quality chronic kidney disease care and the impact of this on the higher rate of heart disease and death seen in African Americans. It will also determine areas of the country where quality of care is poor.

Investigators: This is a multidisciplinary clinical team, Ajay K. Israni, M.D., M.S. - Assistant Professor of Medicine and Adjunct Assistant Professor of Epidemiology at the University of Minnesota will serve as Project Leader of Project 2. He is a nephrologist at Hennepin County Medical Center (HCMC). The Minneapolis Medical Research Foundation (MMRF) manages all of the research grants at HCMC. With the assistance from his co-investigator, Dr. Bertram Kasiske, he will oversee aspects of data collection, management and analysis of data. Bertram Kasiske, M.D. - Professor of Medicine , will serve as Co-Investigator. Kasiske is the Division Chief of Nephrology, Medical Director of Transplantation at HCMC and the Medical Director of Transplantation at the University of Minnesota. He is also the Deputy Director of the United States Renal Data System (USRDS) that is located in MMRF at HCMC. He is also Dr. Israni's mentor on his current NIH K23 grant. He has expertise in the area of kidney disease and extensive experience in leading multi-center studies in chronic kidney disease. Dr. Kasiske will assist Dr. Israni with all aspects of the study. Jon Synder, M.S., Ph.D. - Biostatistician, will serve as Co-Investigator for Project 2. Dr. Snyder is a member of the Chronic Disease Research Group at, MMRF/HCMC and has extensive experience serving as a Biostatistician on NIH and non-federal grants. His Ph.D. thesis utilized the 5% Medicare dataset and he has extensive experience with the USRDS database which will be utilized in this proposal. He also serves as the Biostatistician for the USRDS which will also provide data for this project. Dr. Synder will provide epidemiological and biostatistical support and supervise the data analyst on this project. He will meet with the PI and other research team members to ensure proper analytic strategies are developed and implemented. Eric Weinhandl, M.S. - Statistician (3.60, 4.80. and 5.40 calendar). Eric Weinhandl will serve as the Data Analyst of this project. He has a Masters of Science in biostatistics and experience handling data from large administrative databases such as the USRDS. He has also worked with this team on previous project and continues to do so currently. He will be involved in data cleaning activities and analyzing the data with the supervision from the PI and the Co-Investigators.

Environment: The environment is excellent. The investigative team has access to patients in these clinics and a letter of support signed by both the Executive Director and Medical Director is strong.

Protection of Human Subjects from Research Risks: Appropriate

Inclusion of Women Plan: Appropriate

Inclusion of Minorities Plan: Appropriate

Inclusion of Children Plan: Appropriate

Budget: Reasonable.

CRITIQUE 4 OF THE RESEARCH PROJECT 2

Significance: Chronic Kidney Disease (CKD) is an independent risk factor for atherosclerotic heart disease, and complications present a larger health burden for African Americans. This project aims to determine whether physicians adhere to existing evidence-based guidelines for recognizing CKD as an important ASHD risk factor, and monitor related physiological parameters aggressively.

Approach: The national Medicare 5% sample will be used. The aims are to determine if there are differences in quality of care by ethnic background, determine if quality of CKD care independently explains worse outcomes found in African American medicare patients, and determine characteristics of health service areas in the US with disparities (e.g., low density of providers). The findings of these analyses are to be shared with the community (via the CO/EC) in the last 6 months of the study. Why not share the findings as they are uncovered?

Innovation: Largest CKD study, aimed at validating a more precise algorithm than is currently used to determine CKD with claims data. The algorithm could be used in other administrative dataset to examine CKD patient care. Further, differences in quality of care for African Americans vs. Whites will be examined, and health service areas with disparities uncovered.

Investigators: The ability of the Principal Investigator to conduct this research is unclear. There was no bio-sketch and no description of his research and funding history. However, he is co-author on several papers and his primary Co-Investigator is lead on many of these. He apparently can do this work and has a Co-Investigator that will help with all aspects of this project. Another Co-Investigator will oversee statistical analyses conducted by the statistician.

Environment: The environment is excellent for conducting this research and disseminating findings to have impact (KEEP and CDC focus on CKD at this University). Travel to the CDC is required to secure dataset access.

Human Subjects: A (confidentiality issues)

Risks and Protections: A (Center for Medicare and Medicaid Services and CDC will review any intended publications before submission per their agreements.

Inclusion of women, minorities, children: A, G1,M1, C3 (both, both, adults > 65)

Targeted Recruitment for Clinical Trials: NA

Vertebrate Animals: NA Animal Care and Use: NA

Budget recommendations: Budget seems commensurate with stated activities. It is unclear, however, that three years of funding are required, there is much time allotted for manuscript preparation which could be ongoing with analyses perhaps. This reduction, along with increased start-up time (is nothing happening in the first 2 quarters?) could reduce costs associated with a full year.

THE FOLLOWING RESUME SECTIONS WERE PREPARED BY THE SCIENTIFIC REVIEW OFFICER TO SUMMARIZE THE OUTCOME OF DISCUSSIONS OF THE REVIEW COMMITTEE ON THE FOLLOWING ISSUES:

OVERALL CENTER PROTECTION OF HUMAN SUBJECTS (Resume): ACCEPTABLE

VERTEBRATE ANIMALS (Resume): NOT APPLICABLE

TRAINING IN RESPONSIBLE CONDUCT OF RESEARCH: ACCEPTABLE

RESEARCH PROJECT 1:

PROTECTION OF HUMAN SUBJECTS (Resume): HS protections for children involved needs to be more clearly addressed.

TARGETED RECRUITMENT FOR CLINICAL TRIALS: ACCEPTABLE

INCLUSION OF WOMEN PLAN (Resume): ACCEPTABLE, G1A

INCLUSION OF MINORITIES PLAN (Resume): ACCEPTABLE, M2A

INCLUSION OF CHILDREN PLAN (Resume): ACCEPTABLE, C1A

VERTEBRATE ANIMALS (Resume): NOT APPLICABLE

BIOHAZARDS (IF APPLICABLE) (Resume): NONE

RESEARCH PROJECT 2:

PROTECTION OF HUMAN SUBJECTS (Resume): ACCEPTABLE

TARGETED RECRUITMENT FOR CLINICAL TRIALS: NOT APPLICABLE

INCLUSION OF WOMEN PLAN (Resume): ACCEPTABLE, G1A

INCLUSION OF MINORITIES PLAN (Resume): ACCEPTABLE, M2A

INCLUSION OF CHILDREN PLAN (Resume): ACCEPTABLE, C3A

VERTEBRATE ANIMALS (Resume): NOT APPLICABLE

BIOHAZARDS (IF APPLICABLE) (Resume): NONE

COMMITTEE BUDGET RECOMMENDATIONS:

OVERALL CENTER: See below recommendations for each of its components

ADMINISTRATIVE CORE: Recommended as requested

COMMUNITY CORE: Recommended as requested

RESEARCH TRAINING CORE: The amount for student travel to the Midwest conference seems excessive; the panel recommended it decreasing from $1500 to $1000 per student.

RESEARCH CORE: Recommended as requested

RESEARCH PROJECT 1:
Personnel budget effort appear to be excessive in several categories

- The budgeted effort for the PI is excessive and should be reduced. 20% is suggested
- The budgeted effort for each of the three co-investigators effort is excessive. 5% effort is suggested for Drs. Ahluwalia, An, Hennrik us and Luo
- The budgeted effort for Database Manager is excessive. 10% effort is suggested

RESEARCH PROJECT 2:

- The duration of the project is excessive. A two-year timeline and budget is suggested.
- $22,000 to more than $40,000 per year (55% of salary) in computer services is considered excessive. This should be reduced to $10,000 per year for each of two years.

NOTICE: In 2008 NIH modified its policy regarding the receipt of resubmission (formerly termed amended) applications. Detailed information can be found by accessing the following URL address: http://grants.nih.gov/grants/policy/amendedapps.htm

NIH announced implementation of Modular Research Grants in the December 18, 1998 issue of the NIH Guide to Grants and Contracts. The main feature of this concept is that grant applications (R01, R03, R21, R15) will request direct costs in $25,000 modules, without budget detail for individual categories. Further information can be obtained from the Modular Grants Web site at http://grants.nih.gov/grants/funding/modular/modular.htm

MEETING ROSTER

National Center on Minority Health and Health Disparities Special Emphasis Panel
NATIONAL CENTER ON MINORITY HEALTH AND HEALTH DISPARITIES
Centers of Excellence P60
ZMD1 PA (13) 1
March 18, 2009 - March 20, 2009

CHAIRPERSON

GLOVER, SAUNDRA H., PHD
RESEARCH PROFESSOR
INSTITUTE FOR PARTNERSHIPS TO ELIMINATE
HEALTH DISPARITIES
UNIVERSITY OF SOUTH CAROLINA
COLUMBIA, SC 29210

MEMBERS

ARAR, NEDAL H., PHD
ASSOCIATE RESEARCH PROFESSOR
UNIVERSITY OF TEXAS HSC SAN ANTONIO
DEPARTMENT OF NEPHROLOGY
SAN ANTONIO, TX 782293900

BERDANIER, CAROLYN D., PHD
PROFESSOR EMERITUS
DEPARTMENT OF FOODS AND NUTRITION
UNIVERSITY OF GEORGIA
ATHENS, GA 30602

BERKEL, HANS J. (RETIRED), MD, PHD
PRESIDENT AND CEO OF THE CANCER PREVENTION
INSTITUTE IN DAYTON
PROFESSOR OF EPIDEMIOLOGY
AT THE BOONSHOFT SCHOOL OF MEDICINE
WRIGHT STATE UNIVERSITY
DAYTON, OH 45439

BERKEL, LAVERNE A., PHD
ASSOCIATE PROFESSOR
COUNSELING PSYCHOLOGY AND COUNSELOR
EDUCATION
UNIVERSITY OF MISSOURI AT KANSAS CITY
KANSAS CITY, MO 64110

BINSWANGER, INGRID A., MD
ASSISTANT PROFESSOR
DIVISON OF GENERAL INTERNAL MEDICINE
UNIVERSITY OF COLORADO DENVER
SCHOOL OF MEDICINE
AURORA, CO 80045

BROWN, DANIEL E, PHD
PROFESSOR
DEPARTMENT OF ANTHROPOLOGY
UNIVERSITY OF HAWAII AT HILO
HILO, HI 96720

CAPLAN, LEE , MD
DIRECTOR, DIVISION OF CANCER CONTROL RESEARCH
PREVENTION RESEARCH CENTER
MOREHOUSE SCHOOL OF MEDICINE
ATLANTA, GA 30310

CARROLL, JENNIFER K., MD
RESEARCH ASSISTANT PROFESSOR
DEPARTMENT OF FAMILY MEDICINE
SCHOOL OF MEDICINE AND DENTISTRY
UNIVERSITY OF ROCHESTER
ROCHESTER, NY 14642

DEVON, HOLLI A., PHD
ASSOCIATE PROFESSOR
LOYOLA UNIVERSITY, CHICAGO
NIEHOFF SCHOOL OF NURSING
MAYWOOD, IL 60153

EDWARDS, JOELLEN B., PHD
DIRECTOR, CENTER FOR NURSING RESEARCH
COLLEGE OF NURSING
EAST TENNESSEE STATE UNIVERSITY
JOHNSON CITY, TN 37614

FERRY, ROBERT JR., MD
ASSOCIATE PROFESSOR
PEDIATRIC ENDOCRINE SECTION
UNIVERSITY OF TENNESSEE, MEMPHIS
MEMPHIS, TN 381032800

GRANDISON, DAVID , MD, PHD
ASSOCIATE PROFESSOR
COLLEGE OF PUBLIC HEALTH
UNIVERSITY OF NEBRASKA MEDICAL CENTER
OMAHA, NE 681987810

GRAY, JEREMY R., PHD
ASSISTANT PROFESSOR
DEPARTMENT OF PSYCHOLOGY
& INTERDEPARTMENTAL NEUROSCIENCE PROGRAM
YALE UNIVERSITY
NEW HAVEN, CT 065208205

HELLER, WENDY , PHD
PROFESSOR
DEPARTMENT OF PSYCHOLOGY
DIVISION OF CLINICAL/COMMUNITY
UNIVERSITY OF ILLINOIS
CHAMPAIGN, IL 61820

HU, JENNIFER J., PHD
PROFESSOR AND CO-DIRECTOR
DEPARTMENT OF EPIDEMIOLOGY AND PUBLIC HEALTH
MILLER SCHOOL OF MEDICINE
UNIVERSITY OF MIAMI
MIAMI, FL 33136

JOHANSSON, PATRIK , MD
ASSISTANT RESEARCH PROFESSOR
GEORGE WASHINGTON UNIVERSITY
SCHOOL OF PUBLIC HEALTH & HEALTH SERVICES
WASHINGTON, DC 20037

KHAN, SHAFIQ A., PHD
PROFESSOR AND CHAIR
CENTER FOR CANCER RESEARCH
AND THERAPEUTIC DEVELOPMENT
CLARK ATLANTA UNIVERSITY
ATLANTA, GA 30314

LINTON, MARIGOLD L., PHD
DIRECTOR AMERICAN INDIAN OUTREACH
OFFICE OF THE PROVOST
UNIVERSITY OF KANSAS
LAWRENCE, KS 660451501

LOCKETTE, WARREN E., MD
PROFESSOR OF MEDICINE
DIVISION OF INTERNAL MEDICINE
UNIVERSITY OF CALIFORNIA, SAN DIEGO
SAN DIEGO, CA 92103

LOGUE, EVERETT E., PHD
DIRECTOR
FAMILY PRACTICE CLINICAL RESEARCH
SUMMA HEALTH SYSTEM
AKRON, OH 44309

MANNE, UPENDER , PHD
ASSOCIATE PROFESSOR
DEPARTMENT OF PATHOLOGY
UNIVERSITY OF ALABAMA, BIRMINGHAM
BIRMINGHAM, AL 35294

MAYNARD, SHARON , MD
ASSISTANT PROFESSOR
DIVISION OF RENAL DISEASE AND HYPERTENSION
GEORGE WASHINGTON UNIVERSITY
MEDICAL FACULTY ASSOCIATES
WASHINGTON, DC 20052

MCCABE, MELVINA L, MD
ASSOCIATE PROFESSOR
DEPARTMENT OF FAMILY AND COMMUNITY MEDICINE
1 UNIVERSITY OF NEW MEXICO
ALBUPUERQUE, NM 87131

MCGINN, THOMAS G., MD
PROFESSOR
DEPARTMENT OF MEDICINE
MOUNT SINAI SCHOOL OF MEDICINE
NEW YORK, NY 100296574

MENZEL, NANCY N., PHD
ASSOCIATE PROFESSOR
UNLV SCHOOL OF NURSING
BIGELOW HEALTH SCIENCES
LAS VEGAS, NV 89154

MOY, ERNEST M., MD
MEDICAL OFFICER
CENTER FOR QUALITY IMPROVEMENT
AND PATIENT SAFETY
AGENCY FOR HEALTHCARE RESEARCH AND QUALITY
ROCKVILLE, MD 20860

NIES, MARY A., PHD
CAROL GROTNES BELK ENDOWED CHAIR IN NURSING & PROFESSOR
ADJUNCT PROFESSOR DEPARTMENT OF PUB HLTH SCIS
COLLEGE OF HEALTH AND HUMAN SERVICES
UNIVERSITY OF NORTH CAROLINA AT CHARLOTTE
CHARLOTTE, NC 28223

ODEDINA, FOLAKEMI T, PHD
PROFESSOR AND DIRECTOR
FLORDIA A&M UNIVERSITY
MEMBER & VISITING PROFESSOR
MOFFITT CANCER CENTER
TAMPA, FL 33612

OJO, AKINLOLU O., MD, PHD
PROFESSOR OF MEDICINE
DEPARTMENT OF INTERNAL MEDICINE
UNIVERSITY OF MICHIGAN MEDICAL CENTER
ANN ARBOR, MI 481095364

OPARA, EMMANUEL C., PHD
RESEARCH PROFESSOR & CO-DIRECTOR
ENGINEERING CENTER FOR DIABETES RES & EDUC
PRITZKER INSTITUTE OF BIOMEDICAL SCI & ENGINEERING
ILLINOIS INSTITUTE OF TECHNOLOGY
CHICAGO, IL 60616

SCHNEIDER, TAMERA R., PHD
ASSISTANT PROFESSOR
DEPARTMENT OF PSYCHOLOGY
WRIGHT STATE UNIVERSITY
DAYTON, OH 45435

SCHROY, PAUL C. III, MD
PROFESSOR OF MEDICINE
BOSTON UNIVERSITY SCHOOL OF MEDICINE
DIRECTOR OF CLINCIAL RESEARCH, GI SECTION
BOSTON MEDICAL CENTER
BOSTON, MA 02118

ZOOROB, ROGER , MD
PROFESSOR AND CHAIR
MEHARRY MEDICAL COLLEGE
NASHVILLE, TN 37208

SCIENTIFIC REVIEW ADMINISTRATOR

ATREYA, PRABHA L., PHD
CHIEF, OFFICE OF SCIENTIFIC REVIEW
NATIONAL CENTER ON MINORITY HEALTH
AND HEALTH DISPARITIES
NATIONAL INSTITUTES OF HEALTH
BETHESDA, MD 20892

GRANTS TECHNICAL ASSISTANT

RICHARDSON, ANGELA D.
EXTRAMURAL SUPPORT ASSISTANT
NATIONAL CENTER ON MINORITY HEALTH
AND HEALTH DISPARITIES
NATIONAL INSTITUTES OF HEALTH
BETHESDA, MD 20892

THIGPEN, KIMBERLY
EXTRAMURAL SUPPORT ASSISTANT
NATIONAL CENTER ON MINORITY HEALTH
AND HEALTH DISPARITIES
NATIONAL INSTITUTES OF HEALTH
BETHESDA, MD 20892

Consultants are required to absent themselves from the room during the review of any application if their presence would constitute or appear to constitute a conflict of interest.
